# Antifungal compounds of Chinese prickly ash against drug-resistant *Candida albicans*

**DOI:** 10.1016/j.fochx.2022.100400

**Published:** 2022-07-25

**Authors:** Dan-Yu Ma, Zhao-Jie Wang, Yi-Chi Chen, Zi-Heng Qi, Huan Wang, Yan-Yan Zhu, Xiao-Dong Luo

**Affiliations:** a Key Laboratory of Medicinal Chemistry for Natural Resource, Ministry of Education and Yunnan Province, Yunnan Characteristic Plant Extraction Laboratory, School of Chemical Science and Technology, Yunnan University, Kunming, 650500, PR China; b State Key Laboratory of Phytochemistry and Plant Resources in West China, Kunming Institute of Botany, Chinese Academy of Sciences Kunming, 650201, PR China

**Keywords:** CC, column chromatography of silica gel, DMSO, dimethyl sulfoxide, CFU, colony forming unit, *C. albicans*, *Candida albicans*, MIC, minimum inhibitory concentration, MFC, minimum fungicidal concentration, MTT, 3-(4,5-dimethylthiazole-2-yl)-2,5-diphenyltetrazolium-bromide, PBS, phosphate buffered solution, NMR, nuclear magnetic resonance, SEM, scanning electron microscopy, TLC, thin layer chromatography, FBS, fetal bovine serum, DMEM, Dulbecco's modified Eagle medium, HaCaT, human immortalized keratinocytes, Chinese prickly ash, Chemical ingredients, Anti-fungal activity, Drug-resistant, *Candida albicans*

## Abstract

The leaf of Chinese prickly ash, a unique spice having typical pungent sensation, is a popular food in Southwest China with antipruritic, insecticidal and fungicidal functions, but its bioactive constituents of fungistatic capacity remain unknown. In present investigation, twenty-nine compounds were isolated from leaf of Chinese prickly ash, and their antifungal bioactivity against drug-resistant *Candida albicans* were evaluated *in vitro* and *in vivo*. As a result, three compounds **3**, **10**, **29** showed antifungal bioactivity by damage of the fungal biofilm, and they might recover sensitive of drug resistant *C. albicans* to Fluconazole. Then Chinese prickly ash leaf was proved to be a functional food against fungus for the first time in experiment.

## Highlights

• The antifungal activity of Chinese prickly ash leaf was comprehensively evaluated for the first time.
• Chinese prickly ash leaf extracts were characterized and 40 compounds were identified by MS/MS analysis.
• It has important for the utilization of Chinese prickly ash leaf.

## Graphical abstract

**Figure:**
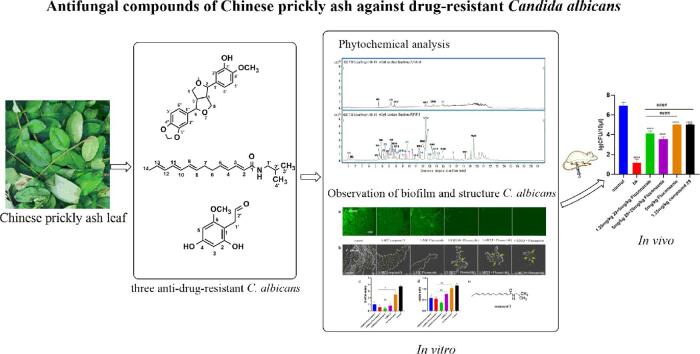

## 1 Introduction

Chinese prickly ash (*Zanthoxylum bungeanum*) is the best known condiment belongs to the *Zanthoxylum* genus in the Rutaceae family which has become a very popular product throughout the world due to its rich variation in aroma and nutritional together with its health benefits (Luo et al., 2021). Originally from warm temperate and subtropical regions of the world, this genus is widely distributed in some parts of southwest China and southeast Asia (Yoro, Franck, Jean, Alassane, & Julien, 2017). The pericarp of this plant is considered a traditional food and used in a wide range of hot pot because of its distinctive aromatic, tongue-numbing sensation, and its pungency (Luo et al., 2021). Chinese prickly ash leaf, the main byproducts of Chinese prickly ash, gain increasing attention due to their unique flavor, wide sources and health-care effects (Ma, Tian, Wang, Huang, & Wei, 2021). In the central part of Yunnan Province, fresh Chinese prickly ash leaf is consumed in salads, cooking, and making tea as a functional food to clear away heat, detoxicate, kill insects, relieve itching (Wu, 2017). Chinese prickly ash leaf contain various types of bioactive compounds, such as flavonoids, amides, lignans, and coumarins, which have anti-inflammatory, antioxidant, antitumor, and antimicrobial properties (Zhang, Luo, Wang, He, & Li, 2014), and then it is consider to be an important resource for the discovery the development of novel functional foods and cosmetics (Wang, 2021). However, in contrast to Chinese prickly ash seeds, Chinese prickly ash leaf is sometimes consumed as fertilizer or discarded, which may not only cause environmental pollution, but also lead to a waste of natural resources.

*C. albicans* is a common commensal fungus in humans that can cause mucosal and deep tissue infections with a mortality rate of up to 35 % (Nett & Andes, 2006). This commensal fungus is able to proliferate when locally or systemically immunosuppressed, of which oropharyngeal candidiasis (OPC) being the most common infection (Millet, Solis, & Swidergall, 2020). The widespread use of Fluconazole has led to resistance of *C. albicans* to Fluconazole, persistent and repeated invasive infection with Fluconazole resistant *C. albicans* increased health care costs and led to higher morbidity and mortality, so need a comprehensive measure in treatment and prevention. Refractory *C. albicans* infection is often related to the formation of biofilms, which is an important mechanism of fungal drug resistance (Hasan et al., 2009, Yu et al., 2011). In the current situation where a number of highly effective and less toxic antifungal drugs was urgently required, combined antifungal therapy has become one of the hotspots (Ahmad, Abida, & Iqbal, 2012). Chinese prickly ash leaf showed antibacterial effect against pathogenic bacteria, *Bacillus cereus*, *B. subtilis* and *Staphylococcus aureus* (Nongmai et al., 2021), but lack of fungistatic capacity and bioactive constituents report.

## 2 Materials and methods

### 2.1 General experimental procedures

All compounds were separated by column chromatography and concentrated by vacuum rotary evaporators (OSB-2200 EYELA). Separate materials included silica gel (200–300 mesh), LiChroprep RP-C18 (40–63 μm), and Sephadex HL-20 (40–70 μm). Partial compounds were purified by an Agilent 1260 Infinity Ⅱ (USA) with a diode array detector using a semipreparative RP-C 18 (250 mm × 10 mm, 5.0 μm). TLC was performed using silica gel GF 254 (HG/T 2354–2010) on a silica gel plate before separation. The isolated compounds were determined by NMR spectroscopy (BRUKER AVANCE III 400 MHz, Switzerland) and processed with Mestrenova software. ESI-MS spectra were recorded on an Agilent 1290/6545 Q-TOF (Agilent, Santa, Clara, USA).

### 2.2 Plant materials

Chinese prickly ash leaf was purchased from Yuxi city, China, in September 2020, and was determined by Professor Li-Xing Zhao of Yunnan University. A specimen was stored in the Key Laboratory of Medicinal Chemistry for Natural Resource, Ministry of Education and Yunnan Province, School of Chemical Science and Technology, Yunnan University, Kunming, P.R. China.

### 2.3 Strains and reagents

*C. albicans* (08030401, drug resistant) was isolated strains presented from the Second People’s Hospital of Yunnan Province. Fluconazole, cyclophosphamide, crystal violet, and MTT were purchased from Macklin Reagents (Shanghai, China). Ethyl acetate, petroleum ether, chloroform, methanol, acetone and acetonitrile were purchased from Tianjin Chemical Reagents Co (Tianjin, China). DMEM, fetal bovine serum, streptomycin, and penicillin were purchased from Gibco, and the human keratinocyte cell (HaCaT) line were generous gifts received from Yunnan University of Traditional Chinese Medicine. All aseptic operations were carried out in a biosafety cabinet (BSC, Thermo Scientific).

### 2.4 Animals experimental conditions

10–12 weeks old male Kunming mice (n = 36, 37 ± 2 g) were purchased from Kunming Medical University. All animals were housed at room temperature (20–26 ℃) and constant humidity (50–70 %) under a 12 h light–dark cycle in a specific-pathogen-free grade laboratory. Mice were allowed to acclimate to their environment for a week before being randomly assigned to experimental groups. The experiment was reviewed and approved by the Institutional Animal Care and Use Committee of Yunnan Institute of Materia Medica.

### 2.5 Characterization of extracts

The fresh Chinese prickly ash leaf (25 kg) was reflux extracted with EtOH (150 L × 3) at 50 ℃ three times (48 h × 3), and the EtOH solvent was evaporated under vacuo to give a crude extract (1.8 kg). The residue was suspended in water and successively partitioned with ethyl acetate (15 L × 3) and water (10 L × 3) to yield the ethyl acetate fraction (510 g) and water fraction (1.26 kg). Then the ethyl acetate fraction was subjected to column chromatography (CC, 200–300 mesh, 2.5 kg) on silica gel eluting with petroleum ether-acetone (from 50:1, 30:1, 15:1, 10:1, 7:1, 3:1, 0:1, 10 L for each ratio) to give eight fractions (Y-1 to Y-8), the detailed method is presented in the Supporting Information.

### 2.6 Identification of compounds from the ethyl acetate fraction by UHPLC-QTOF-MS/MS

The ethyl acetate fraction showed antifungal bioactivity against *C. albicans*, (08030401, drug resistant) and then its constituents was analyzed by an Agilent 1290 UHPLC instrument coupled and G6545B Q-TOF/MS system (Agilent Technologies). The samples were separated by using an Agilent SB-C 18 column (2.1 × 100 mm, 1.8 μm; Agilent Technologies) at a constant temperature (38 ℃). The solvent system consists of water containing 0.1 % (v/v) formic acid (A) and methanol (B) at a flow rate of 0.15 mL/min. The injection volume is 2 μL. The gradient elution was 0–20 min, 5 %-100 % B; 20–25 min, 100 % B. The scanning range in the positive ion mode was *m*/*z* 50–1200. The ESI voltage in the positive ion mode was 3.5 kV. A nebulizing gas of 35 (psig) and a drying gas (7 L/min) were applied for ionization, using nitrogen in the positive ion mode. These conditions were used to identify compounds of ethyl acetate fraction. By comparing fragment ions with the literatures, and the retention time from MS and MS/MS data with the isolated compounds, compounds of ethyl acetate fraction can be identified. By using MS, NMR and comparing with the data of literature to characterize the structure.

### 2.7 Evaluation of antifungal activity *in vitro*

#### 2.7.1 MICs/MFCs determinations

The samples were diluted into seven concentration gradients (128, 64, 32, 16, 8, 4, 2 µg/mL) by the double dilution method in 96-well plates. To ensure the accuracy and reliability of the experiment, each of the samples was repeated six times. Then, 96-well plates were incubated for 8 h at 37 ℃, and read at 600 nm OD. The MIC was reported as the lowest concentration (µg/mL) of the drug and visually inhibited the growth of the organism. The fungi incubated in TSB were used as blank controls, Fluconazole as positive controls, final DMSO concentration<1 %.

After determining the MIC, 10 µL was inoculated onto the supplemented TSA plates containing serial dilutions (1 × MIC, 2 × MIC, 4 × MIC and 8 × MIC), and the plate was incubated at 37 ℃ for 12 h. Each of the samples was repeated three times. The lowest concentration without fungal growth was the MFC.

#### 2.7.2 Checkerboard methods

The microdilution checkerboard method to detect the relationship between compounds and antibiotics synergistic combination of two antifungal agents can be expressed as fractional inhibitory concentration index (FICI), FICI calculated as follows:

$$\text{FICI}=\frac{\mathrm{MIC}\;\text{of drug}A,\;\text{tested in combination}}{\mathrm{MIC}\;\text{of drug}A,\;\text{tested alone}}+\frac{\mathrm{MIC}\;\text{of drug}B,\;\text{tested in combination}}{\mathrm{MIC}\;\text{of drug}B,\;\text{tested alone}}$$

FICI ≤ 0.5, synergistic effect (SY); 0.5 ＜ FICI ≤ 2.0, additive effect (AD); 2.0 ＜ FICI ≤ 4.0, no interaction effect (NI).

Using a twofold dilution series for fungistatic drugs in TSB, the concentration of the two drugs was distributed between 1/16 × MIC and 2 × MIC. Join the fungistatic drugs (50 µL) decrease in the order of concentration in wells of a 96-well plate among the rows of the board, each of the concentrations repeated six times. Then, the same method was used to decrease the compound solution in order of the concentration join it to each column of the 96-well plate. Each of the concentrations was repeated six times, and 100 µL of fungus solution diluted to 10^6^ CFU/mL was added to each well until a final volume of 200 µL was obtained. Then, 96-well plates were incubated for 8 h at 37 ℃ and read at 600 nm OD using a 96-well plate to determine the MIC value of each drug when used in combination. The way is shown in the Supporting Information Fig. S1.

#### 2.7.3 Fungicidal activity was determined by the time-kill curve method

First, samples of different concentrations were added to the 24-well plate so that the final concentrations were 1 × MIC, 2 × MIC and 4 × MIC, and then TSB was added to 0.9 mL in each well. Finally, 100 µL of *C. albicans* (08030401, drug resistant) solution (10^6^ CFU) was added to obtain a final volume (1 mL). 1 % (v/v) DMSO was used as control, each of the samples was repeated three times. The 24-well plates in a 37 ℃ constant temperature incubator, and at 0, 1, 2, 3, 4, and 6 h, 10 μL of culture solution was removed from each group, diluted to different multiples with sterile PBS solution and then coated on TSA solid. After culturing for 24 h, the number of colonies grown at the appropriate dilution was recorded, and the corresponding time point for culturing the concentration of *C. albicans* (08030401, drug resistant) in the base (CFU/mL) was recorded.

#### 2.7.4 Fungal biofilms activity and scanning electron microscope (SEM)

As described previously, *C. albicans* (08030401, drug resistant) solution (10^6^ CFU) was grown in 96-well plates at 37 ℃ for 24 h to allow biofilm formation. Then, the medium was removed from each well and washed three times with sterile PBS to remove planktonic fungi that had not formed biofilms. Finally, different drugs diluted with TSB were added and incubated at 37 °C for 24 h. All treatments were repeated 5 times. The quality of the biofilm was assessed using the crystalline violet assay (OD at 600 nm) and the viable cells in the formed biofilm were determined using the MTT assay (OD at 570 nm).

SEM was used to analyze the biofilm morphology as described, keeping concentration samples into 24-well plates to make the final concentrations 1/2 × MIC, 1 × MIC, and 2 × MIC. Each of the samples was repeated three times at 37 ℃ for 3 h, and centrifuged (3500 rpm, 20 min). The supernatant was removed, washed three times with PBS, then fixed with 4 % paraformaldehyde for 24 h. Finally, the samples were dehydrated with gradient concentrations of ethanol (30 % for 10 min, 50 % for 10 min, 70 % for 10 min, 90 % for 10 min, and 100 % for 10 min), dried, and sprayed with gold. The samples were observed and photographed under SEM.

#### 2.7.5 Cell culture and cell viability assay

The cytotoxic activities of isolated compounds were evaluated by the MTT method. The HaCaT cells were cultured in DMEM supplemented with 10 % heat-inactivated FBS, 100 U/mL benzylpenicillin/streptomycin at 37 °C in a humidified incubator with 5 % CO_2_. In order to determine the influence of additive to HaCaT cells, they were seeded in 96-well plates and cultured for 48 h with MIC of the tested compound alone or in combination with Fluconazole, or DMSO (control). Then, 10 % MTT solution (5 mg/mL) was added and incubated at 37 °C for 4 h. The absorbance was measured at 490 nm by a microplate reader.

### 2.8 Antifungal activity *in vivo*

Model groups for a total of 36 samples (weighing 37 ± 2 g each sample) were randomly divided into six groups, with six replicates for each group: blank group, control group, positive drug group (5 mg/kg of Fluconazole), high-dose group (5 mg/kg of compound **29** and 20 mg/kg of Fluconazole), low-dose group (1.25 mg/kg of compound **29** and 5 mg/kg of Fluconazole), and single compound group (1.25 mg/kg of compound **29**). Six groups of mice established hypo-immunity by intraperitoneal injection of cyclophosphamide (30 mg/kg) one day in advance. Except for the blank group, the remaining five groups were treated with sterile cotton swabs dipped in *C. albicans* (08030401, drug resistant) (10^6^ CFU) and kept on the oral mucosa of the mice for 1 min. Different drug treatments were administered at 3, 24 and 27 h after the start of infection, and the control group was treated with the same amount of normal saline, and the results were tested on day 3. Using a sterile cotton swab of the applicator mouse mouth for 1 min, the cotton tip was cut off and placed into an EP tube containing 0.99 mL of PBS, the EP tube was vortexed for 1 min and 100 µL of the mixture was serially diluted 10-fold with PBS. 10 µL of the mixture was transferred to TSA plates, and each sample was repeated three times. After incubation at 37 °C for 24 h, the colonies on the TSA plates were enumerated. All operations were carried out in a biosafety cabinet (BSC).

## 3 Results and discussion

### 3.1 Isolation of compounds from the Chinese prickly ash leaf

29 known compounds were isolated and identified as (-)qin bun amide A (**1**) (Tian et al., 2016), qin bun amide B (**2**) (Tian et al., 2016), *γ*-sanshoöl (**3**) (Yasuda, Takeya, & Itokawa, 1981), (-)-asarinin (**4**) (Joonseok et al., 2010), (-)-kobusin (**5**) (Abe, 1974), (-)-xanthoxylol-4′-*O*-*γ*,*γ*-dimethylallyl ether (**6**) (Bastos, Gottlieb, Sarti, & Filho, 1996), (+)-magnolin (**7**) (Hua, Ah, Hee, Ryang, & Ho, 2004), (+)-eudesmin (**8**) (Latip, Hartley, & Waterman, 1999), planispine A (**9**) (Liu, Min, Huang, & Chen, 2019), (+)-piperitol (**10**) (Terreaux, Maillard, Hostettmann, Lodi, & Hakizamungu, 2010), nodakenetin (**11**) (Saeed & Sabir, 2008), bargapten (**12**) (Li, 2009), imperatorin (**13**) (Wang et al., 2008), demethylsuberosin (**14**) (Masuda, Takasugi, & Anetai, 1998), pubinernoid A (**15**) (Huang et al., 2006), linalyl-*β*-glucopyranoside (**16**) (Omar, Noman, Mohamed, Altuntas, & Demirtas, 2018), (3S,5R,6S,7E)-5,6-epoxy-3-hydroxy-7-megastigmen-9-one (**17**) (D”Abrosca et al., 2004), (3R,6R,7E)-3-hydroxy-4,7-megastigmadien-9-one (**18**) (D”Abrosca et al., 2004), linalyl-*O*-*β*-*d*-glucoside (**19**) (Sang, Min, Lee, Kim, & Kang, 2007), (-)epicatechin (**20**) (Zhou & Yang, 2000), quercetin (**21**) (Zhou & Yang, 2000), 4′-*O*-methyl catechin (**22**) (Docampo-Palacios et al., 2020), 1*H*-indole-3-carbaldehyde (**23**) (Wang, Yang, Mei, & Yang, 2013), *N*^14^-formyldihydrorutaecarpine (**24**) (Yang, Zhang, & Hu, 2008), 2,6,2′,6′-tetramethoxy-4,4′-bis(2,3-epoxy-1-hydroxypropyl)biphenyl (**25**) (Shiow-Hwa et al., 2001), cinnamic acid (**26**) (Yang, Zhao, Ren, Mei, & Liang, 2003), loliolide (**27**) (Willuhn & Westhaus, 1987), 4-hydroxy-3-methoxy-benzaldehyde (**28**) (Ito et al., 2001), 2, 4-dihydroxy-6-methoxy acetophenone (**29**) (Singh, Pathak, & Agrawal, 1997) by comparison with literatures.

### 3.2 Phytochemical analysis

**Fig. 1:**
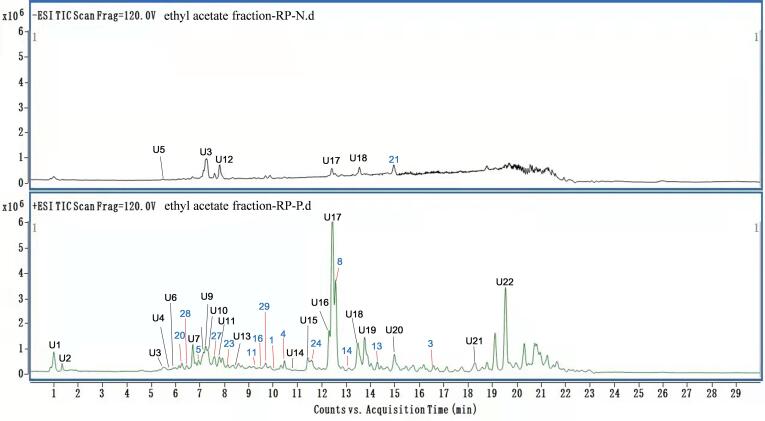
Total ion chromatograms (TIC) of the ethyl acetate fraction in − ESI and + ESI. The black numbered peaks (**U1**–**U22**) were not isolated compounds and the blue numbered peaks were isolated compounds.

Forty compounds were identified from the ethyl acetate fraction of Chinese prickly ash leaf (Fig. 1)., and the main ingredients can be formally divided into the five types, amides, flavonoids, lignans, coumarin and volatile oils. The secondary mass spectrometry analysis of the compound gave a series of fragments, which can be used as a reference for identification of the same type of compound by LC-MS. In the positive ion mode and negative ions mode, the peaks were identified by comparison with standard compounds and MS data statistical analysis in Table 1.

**Table 1:** Qualitative Analysis Constituents of Chinese prickly ash leaf.

Peak	tR(min)	Molecular formula	Molecular ion	Compounds	MS/MS	References In S1
**U1**	1.014	C_15_H_17_NO_3_	M + H^+^ 260.1281	Araliopsine	260.0, 231.1	(Gregory et al., 2001)
**U2**	1.365	C_13_H_10_O_5_	M + H^+^ 247.0601	Isopimpinellin	247.0, 229.0, 147.1, 90.9	(Wang et al., 2021)
**U3**	5.517	C_16_H_18_O_9_	M + H^+^ 355.1024	Chlorogenic acid	355.1, 163.0	(Vinod et al., 2016)
**U4**	5.754	C_9_H_8_O_3_	M + H^+^ 165.0541	Hydroxycinnamic acid	294.8, 163.1, 148.1	(Anna et al., 2015)
**U5**	5.773	C_27_H_32_O_15_	M−H^+^ 595.1668	Eriocitrin	595.1, 287.0, 147.0	(Noha et al., 2017)
**U6**	5.974	C_9_H_8_O_4_	M + H^+^ 181.0495	Caffeic acid	181.0, 163.0, 145.0, 135.0	(Wang et al., 2021)
**20**	6.296	C_15_H_14_O_6_	M + H^+^ 291.0863	(-) Epicatechin	292.2, 139.0	(Vinod et al., 2016)
**28**	6.612	C_8_H_8_O_3_	M + H^+^ 153.0546	4-hydroxy-3-methoxy-benzaldehyde	191.1, 173.0, 153.1	(Chen et al., 2020)
**U7**	6.705	C_20_H_23_NO_4_	M + H^+^ 342.1704	*N*-(4′-methoxy-phenethyl)-3,4-dimethoxy-cinnamamide	342.1, 135.0, 163.0	(Wang et al., 2021)
**5**	6.927	C_21_H_22_O_6_	M + H^+^ 371.1489	(-)-Kobusin	469.0409.1, 371.1, 353.1, 335.1, 233.1	(Vinod et al., 2016)
**U8**	7.093	C_21_H_20_O_10_	M + H^+^ 433.1129	Isovitexin	455.1, 433.1, 415.1, 397.1, 367.1, 337.1	(Vinod et al., 2016)
**U9**	7.344	C_16_H_25_NO_3_	M + H^+^ 280.1907	Zanthoxylumamide A	280.1, 262.1, 182.1, 143.0	(Wang et al., 2021)
**U10**	7.378	C_19_H_19_NO_4_	M + H^+^ 326.1387	Armatamide	326.1, 175.0, 145.1, 135.0	(Wang et al., 2021)
**27**	7.452	C_11_H_16_O_3_	M + H^+^ 197.1167	Loliolide	197.1, 163.0, 153.1, 111.1	(Fei et al., 2009)
**U11**	7.544	C_10_H_12_O	M + H^+^ 149.0961	Estragole	149.0, 133.0	(Hoi-Seon 2016)
**U12**	7.899	C_28_H_34_O_15_	M−H^+^ 609.1825	Hesperidin	610.2, 600.9, 565.1	(Roja et al., 2020)
**23**	8.065	C_9_H_7_ON	M + H^+^ 146.0601	^1^H-indole-3-carbaldehyde	146.0, 116.0,77.1	(Wang et al., 2013)
**U13**	8.341	C_25_H_30_O_6_	M + H^+^ 427.2115	Planispine-A	427.1, 341.2	(Vinod et al., 2016)
**11**	9.545	C_14_H_14_O_4_	M + H^+^ 247.0968	Nodakenetin	247.1, 213.2, 187.0, 131.0, 59.0	(Saeed & Sabir, 2007)
**16**	9.695	C_16_H_28_O_6_	M + Na^+^ 339.1958	Linalyl-*β*-glucopyranoside	339.1, 215.0	(Sang Un et al., 2007)
**29**	9.869	C_9_H_10_O_2_	M + H^+^ 183.0649	2,4-dihydroxy-6-methoxyacetophenone	183.1, 166.0	(Ke et al., 2020)
**1**	10.014	C_18_H_31_O_4_N	M + H^+^ 326.2326	(-) qin bun amide A	348.2, 326.1	(Ke et al., 2020)
**4**	10.473	C_20_H_18_O_6_	M + H^+^ 355.1176	(-)-Asarinin	355.1, 319.1,135.1	(Vishal et al., 2014)
**U14**	10.708	C_11_H_6_O_3_	M + H^+^ 187.039	Psoralen	187.0,131.1	(Vinod et al., 2016)
**U15**	11.439	C_12_H_8_O_4_	M + H^+^ 217.0495	Bergapten	217.1, 202.1, 174.1, 146.1	(Yoro et al., 2017)
**24**	11.784	C_19_H_15_N_3_O_2_	M + H^+^ 318.3015	*N*^14^-formyldihydrorutaecarpine	318.3, 288.1	(Yang et al., 2008)
**U16**	12.267	C_10_H_16_	M + H^+^ 137.1325	*α*-phellandrene	137.0,121.1, 105.0, 77.0	(Luo et al., 2003)
**U17**	12.419	C_16_H_25_NO_2_	M + H^+^ 246.1958	Alpha-hydroxy-sanshool	264.1, 246.1, 147.1 107.0	(Wang et al., 2021)
**8**	12.616	C_22_H_26_O_6_	M + H^+^ 387.1802	(+)-Eudesmin	387.1, 369.0, 351.1, 201.1	(Kumar et al., 2014)
**14**	13.195	C_14_H_14_O_3_	M + H^+^ 231.1008	demethylsuberosin	253.0, 231.1	(Masuda et al., 1998)
**U18**	13.249	C_15_H_16_O_4_	M + H^+^ 261.1121	2′-Methoxyaucuparin	261.1, 246.1, 229.0, 201.1	(Wang et al., 2021)
**U19**	13.756	C_18_H_27_NO_2_	M + H^+^ 290.2115	Galma-hydroxy-sanshool	290.2, 272.2, 165,1	(Wang et al., 2021)
**13**	14.321	C_16_H_14_O_4_	M + H^+^ 271.0956	imperatorin	293.0, 271.1, 270.0	(Takahiro et al., 1998)
**U20**	15.001	C_16_H_25_NO	M + H^+^ 248.2009	*α*-sanshoöl	270.1, 248.1, 175.1, 154.1, 149.1, 133.1	(Vinod et al., 2016)
**21**	15.037	C_15_H_10_O_7_	M−H^+^ 301.0354	quercetin	603.0, 301.1, 207.8	(Roja et al., 2020)
**3**	16.462	C_18_H_27_NO	M + H^+^ 274.2157	*γ*-sanshoöl	296.2, 274.2	(Ke et al., 2020)
**U21**	18.232	C_27_H_32_O_15_	M + H^+^ 597.1814	Eriodictyol-7-O-rutinoside	597.1, 415.3	(Wang et al., 2021)
**U22**	19.527	C_16_H_27_NO_3_	M + H^+^ 282.2064	Zanthoxylumamide C	282.2, 265.2	(Wang et al., 2021)

### 3.3 Antifungal activity *in vitro*

#### 3.3.1 Determination of MICs and MFCs

The antifungal activity of 29 compounds against *C. albicans* (08030401, drug resistant) were test by the microdilution broth susceptibility assay, the results were the MICs of compounds **2**, **3**, **10**, **25**, **26**, **28** and **29** were 32 µg/mL, and the MIC of compounds **1**, **9**, **11**, **20** and **21** were 64 µg/mL. The detailed results shown in the Supporting Information Table S1.

Seven compounds with MIC of 32 µg/mL were tested for synergistic effects with Fluconazole used a checkerboard method. (Table 2**)**.

**Table 2:** Antifungal activity against *C. albicans* (drug resistant).

Strain	Compounds	Alone		Combined		FICI	Remark
		MICs compound (*μg*/mL)	MICs Fluconazole (*μg*/mL)	MICs compound (*μg*/mL)	MICs Fluconazole (*μg*/mL)		
	**2**	32	128	16	32	＞0.5	AD
	**3**	32	128	8	32	≤ 0.5	SY
	**10**	32	128	8	32	≤ 0.5	SY
*C. albicans (08030401)* ***albicans*(08030401)**	**25**	32	128	16	32	＞0.5	AD
	**26**	32	128	16	32	＞0.5	AD
	**28**	32	128	16	32	＞0.5	AD
	**29**	32	128	8	32	≤ 0.5	SY

Additive, (AD), synergistic, (SY), no interaction, (NI).

To describe the combined effects of the seven compounds and antibiotics, the checkerboard experiments were performed followed by FICI numerical calculations and interval definitions. The results of the checkerboard experiment showed that the combination of three compounds (**3**, **10**, **29**) with Fluconazole had a synergistic effect on *C. albicans* (08030401, drug resistant) (FICI ≤ 0.5). The MIC of Fluconazole and compounds (**3**, **10**, **29**) against *C. albicans* (drug resistant) was reduced to a quarter after the combination. In subsequent experiments, the MIC of the compounds in combination with Fluconazole was further investigated including the antifungal pattern *in vivo* and *in vitro*.

#### 3.3.2 The time-kill curve technique

**Fig. 2:**
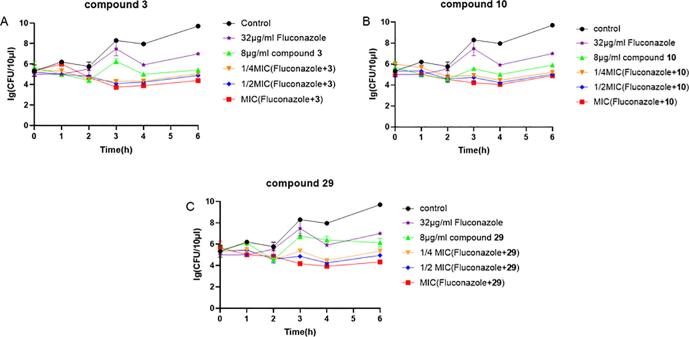
Time-kill kinetics of compound **3** (A), compound **10** (B), and compound **29** (C) alone and combined with Fluconazole against resistant *C. albicans.*

To gain insight into the fungistatic mechanism of antibiotic combinations compound, fungistatic and synergistic effects of the tested antibiotic combinations compound were assessed by using the time-kill curve technique. This experiment can judge whether the combination of compounds and antibiotics has concentration-dependent and time-dependent properties, and can further judge whether the compound is a fungicidal compound or a fungistatic compound. Adding 1/4 × MIC, 1/2 × MIC, 1 × MIC (compounds **3**, **10**, **29** + Fluconazole), *C. albicans* (08030401, drug resistant) were in a zero-growth state within 6 h. *C. albicans* began to grow after 3 h after treatment with 1/4 MIC compound alone and Fluconazole, and the control group continued to grow 2 h later. The results indicated that Fluconazole resistant *C. albicans* could be recovered its sensitive by three compounds (Fig. 2**)**.

#### 3.3.3 Antibiofilm activity and SEM.

**Fig. 3:**
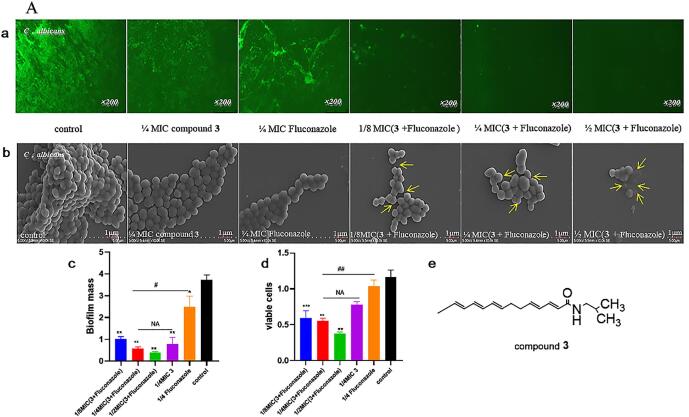
*C. albicans* were treated with compound **3** and Fluconazole alone or in combination. (a) Biofilms were observed under a fluorescence microscope at 200×. (b) SEM photography of *C. albicans*. The yellow arrows represent the observed morphological changes. (c) Biofilms were detected by the MTT method. (d) Biofilms were detected by the crystal violet method. (e) The chemical structure of compound. Values were mean ± SD. *P < 0.05, **P < 0.01, ***P < 0.001 versus control. ^#^P < 0.05, ^##^P < 0.01.

Biofilms produced by fungal is one of the reasons for the development of disease pathogenicity and drug resistance. In order to explore the most possible antifungal mechanism of compounds, fluorescence microscopy imaging and SEM method were used. The detection results are shown in Fig. 3 and Figs. S2–S3*.*

In the Fig. 3**,** compound **3** and Fluconazole were used alone or combined to explore the antibiofilm activities of C. albicans (08030401, drug resistant). visualization of C. albicans biofilms under fluorescence microscopy (a) the control showed significant clumpy green fluorescence indicating that there was more biofilm. This part of the biofilm was visible microscopically when the compounds and fluconazole were used alone compared to the control group. As the concentration of the (compounds + Fluconazole) group increases, the green fluorescence gradually decreased to almost nothing, this implied that fluconazole alone could not destroy biofilm while in combination with the compound could destroy biofilm. Meanwhile, biofilms and viable cells were quantified by the crystal violet staining method or MTT staining method. The results showed that compared with control the (compounds + Fluconazole) group at 1/4 MIC and 1/2 MIC significantly reduced the amount of biofilm (c) and viable cells (d). When the compounds and Fluconazole were used alone, there was also a small clearance effect on biofilms and viable cells. Therefore, it was concluded that the compounds (**3**, **10**, **29**) in combination with Fluconazole cleared the biofilm of C. albicans.

SEM (b) showed that the surface of control group C. albicans structure was found to be smooth and bright without obvious damage. However, in the compounds and Fluconazole were used alone group, the number of fungi in the same field of vision was slightly lower than that in the control group, and the external morphology of fungi was trifling changed. And in the 1/8 MIC (compounds + Fluconazole) group, the structure of fungi began to rupture. In the 1/2 MIC (compounds + Fluconazole) group, the number of fungi decreased further, the external morphology of most of the fungi changed significantly, the fungi no longer presented sphericity, and the structure appeared to shrink and rupture. The results showed that the compounds (**3**, **10**, **29**) in combination with Fluconazole not only disrupt biofilms but also rupture cell structures.

#### 3.3.4 Cytotoxic assay

The cytotoxicity of compounds (**3**, **10**, **29**) and Fluconazole was determined using the HaCaT cells by the MTT method. The relative viability of the cells incubated with the extract was calculated taking as reference the untreated cultures (control). The results indicated that 128 μg/mL Fluconazole showed 48.69 % cell viability, and the dosage was reduced when combined with the compounds (**3**, **10**, **29**), the toxicity was significantly reduced, and no cytotoxic effects of three compounds (**3**, **10**, **29**), as shown as shown in Fig. S4**.**

### 3.4 Antifungal activity *in vivo*

**Fig. 4:**
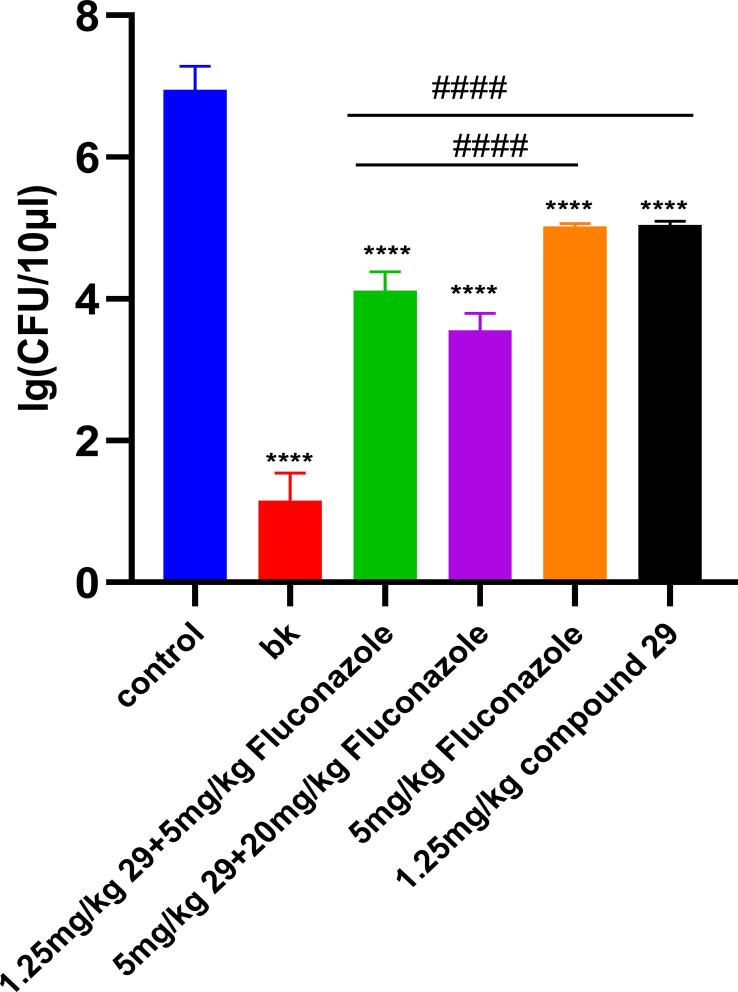
The combination of compound **29** and Fluconazole inhibited *C. albicans* in mice. (Oral mucosa mice were infected with *C. albicans* and treated with positive drug group (5 mg/kg of Fluconazole), high-dose group (5 mg/kg of compound **29** and 20 mg/kg of Fluconazole), low-dose group (1.25 mg/kg of compound **29** and 5 mg/kg of Fluconazole), single compound group (1.25 mg/kg of compound **29**), control group and blank group (wiped with saline). Data were expressed as means ± SD for all mice. Six replicates for each group.) Values were mean ± SD. ****P < 0.0001 versus control. ^#^P < 0.05, ^##^P < 0.01, ^###^P < 0.001, and ^####^P < 0.0001.

In humans, the most common *Candida* species found in both healthy oral mucosa and in Oral candidiasis is *C. albicans* due to its adherence properties and greater level of pathogenicity. Therefore, established a mouse model of oral infection of *C. albicans* (08030401, drug resistant) who’s oral can be treated by compound and Fluconazole to evaluate whether the compound and fluconazole alone or in combination can effectively remove oral infection of *C. albicans*. As a result, the *C. albicans* growth was significantly inhibited in the co-administration group compared to the alone administration group. Similar to the findings *in vitro*, after being with *C. albicans* for three days, in the control group (wiped with normal saline), *C. albicans* in the oral cavity grew to 10^7^ CFU in mice, and *C. albicans* in the give different doses drugs mice were between 10^3^ and 10^5^ CFU, compared with control the inhibition rate were 40.76 % (1.25 mg/kg compound **29** and 5 mg/kg Fluconazole), 48.82 % (5 mg/kg compound **29** and 20 mg/kg Fluconazole), 27.5 % (1.25 mg/kg compound **29**), 27.79 % (5 mg/kg Fluconazole), respectively (Fig. 4). The results further supported that Fluconazole resistant *C. albicans* could be recovered its sensitive by three compounds *in vivo*.

## 4 Conclusions

It was the first time to examine antifungal activity of Chinese prickly ash leaf*,* in which three bioactive compounds (**3**, **10**, **29**) showed significant antifungal activity against drug-resistant *C. albicans*. Moreover, three compounds disrupted preformed biofilms of drug-resistant *C. albicans*, and they had significant synergistic effect combined with fluconazole. The finding extended the potential value of Chinese prickly ash leaf as a functional food to reduce the use of antibiotics in treating drug-resistance fungi.

## Declaration of Competing Interest

The authors declare that they have no known competing financial interests or personal relationships that could have appeared to influence the work reported in this paper.
