# Supplementary material for: Antifungal compounds of Chinese prickly ash against drug-resistant Candida albicans
Source: Food Chem X. 2022 Jul 25;15:100400. doi: 10.1016/j.fochx.2022.100400 (PMC9532736; doi:10.1016/j.fochx.2022.100400)
Supplement: Supplementary data 1 [file mmc1.pdf]

Supporting information

Antifungal compounds of Chinese prickly ash against drug-resistant *Candida albicans*

Dan-Yu Ma [a, 1], Zhao-Jie Wang [a, 1], Yi-Chi Chen [a], Zi-Heng Qi [a], Huan Wang [a], Yan-Yan Zhu [a], Xiao-Dong Luo [a, b*]

[a] *Key Laboratory of Medicinal Chemistry for Natural Resource, Ministry of Education and Yunnan Province, Yunnan Characteristic Plant Extraction Laboratory, School of Chemical Science and Technology, Yunnan University, Kunming, 650500, PR China*

[b] *State Key Laboratory of Phytochemistry and Plant Resources in West China, Kunming Institute of Botany, Chinese Academy of Sciences Kunming, 650201, P. R. China*

Directory

Antifungal compounds of Chinese prickly ash against drug-resistant *Candida albicans* 1
1.NMR of compounds from Chinese prickly ash leaf 1
2.Separation methods of the compounds...18
3.Determination of MICs and MFCs 20
4.Antibiofilm activity and SEM. 21
5.Cytotoxic assay 23
6. S1. References 23

1.NMR of compounds from Chinese prickly ash leaf

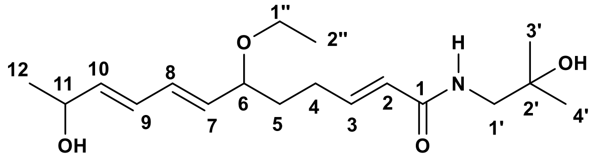

Compound **1**

Qin bun amide A **(1)**: $C_{18}H_{31}NO_4$; ^{1}H-NMR (400 MHz, MeOH) δ_H: 7.12 (1H, dt, *J* = 15.1, 7.9 Hz, H-3), 6.77 (1H, dd, *J* = 15.1, 10.7 Hz, H-9), 6.25 (1H, d, *J* = 14.58 Hz, H-2), 6.23 (1H, dd, *J* = 14.25, 10.8 Hz, H-8), 5.52 (1H, dd, *J* = 14.52, 7.9 Hz, H-7), 3.74 (overlapped, H-6), 3.53 (1H, dq, *J* = 16.5, 7.1 Hz, H-1''), 3.25 (1H, dq, *J* = 16.5, 17.0 Hz, H-1''), 2.31 (2H, t, *J* = 7.1 Hz, H-4), 1.71 (1H, dt, *J* = 14.1, 7.6 Hz, H-5), 1.61 (1H, dt, *J* = 14.1, 7.6 Hz, H-5), 1.23 (1H, d, *J* = 6.23 Hz, H-12), 1.17 (3H, s, H-3'), 1.17 (3H, s, H-4'), 1.14 (1H, t, *J* = 7.0 Hz, H-2''); ^{13}C-NMR (100 MHz, MeOH) δ_C: 14.2 (q, C-2''), 22.1 (q, C-12), 25.8 (q, C-3'), 27.6 (q, C-4'), 27.6 (t, C-4), 34.0 (t, C-5), 49.6 (t, C-1'), 63.4 (t, C-1''), 67.4 (d, C-11), 70.2 (s, C-2'), 79.3 (d, C-6), 123.4 (d, C-2), 128.0 (d, C-9), 132.0 (d, C-8), 133.2 (d, C-7), 137.7 (d, C-10), 144.0 (d, C-3), 167.6 (s, C-1); ESI-MS: *m/z* 348.1 $[M+Na]^+$.

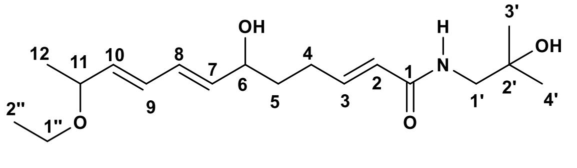

Compound **2**

qin bun amide B **(2)**: $C_{18}H_{31}NO_4$; ^{1}H-NMR (400 MHz, MeOH) δ_H: 6.80 (1H, dt, *J* =

15.1 Hz, 6.8 Hz, H-3), 6.26 (1H, dd, J = 15.1, 10.1 Hz, H-8), 6.22 (1H, dd, J = 15.1, 10.1 Hz, H-9), 6.00 (1H, d, J = 14.8 Hz, H-2), 5.71 (1H, dd, J = 14.2, 6.1 Hz, H-7), 5.56 (1H, dd, J = 14.2, 7.4 Hz, H-10), 4.33 (1H, m, H-6), 3.82 (1H, m, H-11), 3.49 (1H, m, H-1''), 3.37 (1H, m, H-1''), 3.04 (1H, d, J=6.9 Hz, H-1'), 2.28 (1H, m, H-4), 2.06 (1H, m, H-5), 1.89 (1H, m, H-5), 1.45 (2H, s, H-3', H-4'), 1.21 (3H, d, J=6.4 Hz, H-12), 1.17 (6H, t, J=7.0 Hz, H-2''); ^{13}C-NMR (100 MHz, MeOH) δ_C: 14.2 (d, C-2), 22.1 (q, C-12), 25.8 (q, C-3'), 27.6 (q, C-4'), 27.6 (t, C-4), 34.0 (t, C-5), 49.6 (t, C-1'), 63.4 (t, C-1''), 67.4 (d, C-11), 70.2 (s, C-2'), 79.3 (d, C-6), 123.4 (d, C-2), 128.0 (d, C-9), 132.0 (d, C-8), 133.2 (d, C-7), 137.7 (t, C-1'), 144.0 (d, C-3), 167.6 (s, C-1); ESI-MS: *m/z* 348.1 [M+Na]$^+$.

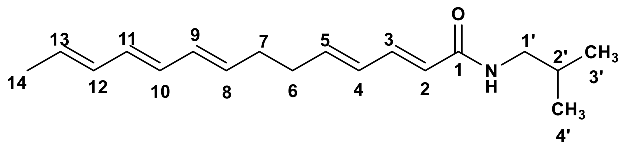

Compound **3**

***γ-sanshoöl* (3)**: $C_{18}H_{27}NO$; ^{1}H-NMR (400 MHz, CDCl$_3$) δ_H: 7.18 (1H, dd, J = 14.9, 10.5 Hz, 3 H), 5.96-6.30 (6H, m), 5.77 (1H, d, J = 14.9 Hz, 2-H), 5.7 (1H, m), 5.25-5.56 (2H, m), 3.16 (2H, dd, J = 6.5 Hz, 1'-H), 2.29 (4H, m, 6-H, 7-H), 1.76 (4H, m, 14-H, 2'-H), 1.92 (6H, d, J = 6.8 Hz,); ^{13}C-NMR (100 MHz, CDCl$_3$) δ_C: 18.3 (q, C-14), 20.1 (q, C-3'), 27.0 (t, C-7), 28.6 (d, C-2'), 32.9 (t, C-6), 46.9 (t, C-1'), 122.2 (d, C-2), 125.3 (d, C-10), 128.7 (d, C-4), 129.4 (d, C-8), 129.9 (d, C-9), 130.0 (d, C-13), 131.8 (d, C-12), 133.3 (d, C-11), 141.0 (d, C-3), 141.8 (d, C-5), 166.3 (s, C-1); ESI-MS: *m/z* 274.2 [M+H]$^+$.

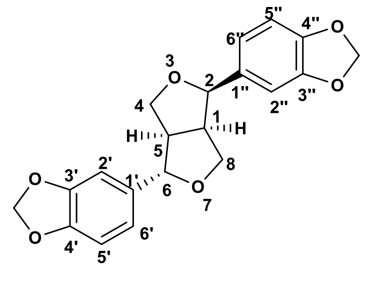

Compound **4**

(-)-Asarinin **(4)**: $C_{20}H_{18}O_6$; ^{1}H-NMR (400 MHz, $CDCl_3$) δ_H: 7.07-6.69 (6H, m, H-2', H-2'', H-5', H-5'', H-6', H-6''), 5.98 (2H, s, O-CH_2O), 5.97 (2H, s, -OCH_2O-), 4.85 (6H, d, J = 5.3 Hz, H-6), 4.42 (1H, d, J = 7.2 Hz, H-2), 4.12 (1H, d, J = 9.5 Hz, H-8β), 3.85(2H, m, H-8α, H-4 α), 3.32 (2H, m, H-5, H-4β), 2.88 (1H, m, H-1); ^{13}C-NMR (100 MHz, $CDCl_3$) δ_C: 50.1 (d, C-1), 54.6 (d, C-5), 69.6 (t, C-8), 70.9 (t, C-4), 82.0 (d, C-2), 87.6 (d, C-6), 100.9 (t, O-CH_2O), 101.0 (t, O-CH_2O), 106.4 (d, C-2'), 106.5 (d, C-2''), 108.1 (d, C-5', C-5''), 118.6 (d, C-6'), 119.6 (d, C-6''), 132.2 (s, C-1'), 135.1 (s, C-1''), 146.5 (s, C-3'), 147.2 (s, C-4''), 147.6 (s, C-4'), 147.9 (s, C-3''); ESI-MS: *m/z* 355.0 $[M+H]^+$.

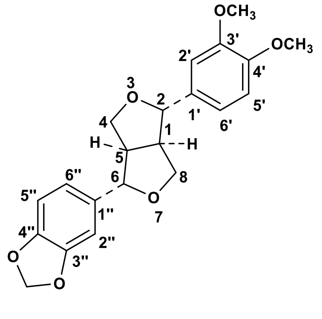

Compound **5**

(-)-Kobusin **(5)**: $C_{21}H_{22}O_6$; ^{1}H-NMR (400 MHz, $CDCl_3$) δ_H: 7.12-6.34 (6H, m, aromatic), 5.95 (2H, s, -OCH_2O), 4.24 (2H, dd, J = 9.0, 4.0 Hz, H-4b/8b), 3.91 (2H, m,

H-4a/8a), 3.89 (3H, s, OCH_3), 3.87 (3H, s, -OCH_3), 3.08 (2H, m, H-1/5); ^{13}C-NMR (100 MHz, $CDCl_3$) δ_C: 54.1 (d, C-1), 54.3 (d, C-5), 55.9 (q, -OCH_3), 55.9 (q, -OCH_3), 71.6 (t, C-8), 71.7 (t, C-4), 85.7 (d, C-6), 85.8 (d, C-2), 101.0 (q, -OCH_3), 106.5 (d, C-2''), 108.2 (d, C-5''), 109.2 (d, C-2''), 111.0 (d, C-5'), 118.2 (d, C-6'), 119.3 (d, C-6''), 133.5 (s, C-1'), 135.1 (s, C-1''), 147.1 (s, C-4''), 147.9 (s, C-3''), 148.6 (s, C-4'), 149.2 (s, C-3'); ESI-MS: *m/z* 371.0 $[M+H]^+$.

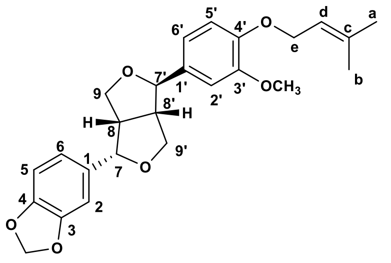

Compound **6**

(-)-xanthoxylol-4'-O-γ,γ-dimethylallyl ether **(6)**: $C_{25}H_{28}O_6$; ^{1}H-NMR (400 MHz, $CDCl_3$) δ_H: 7.3 (1H, s), 6.74-6.99 (2H, m), 5.96 (1H, s, -OCH_2O), 5.51 (1H, t, 6.8, H b), 4.84 (1H, d, J = 5.3 Hz, H-2), 4.57 (1H, d, J = 6.7 Hz, Hc), 4.42 (1H, d, J = 7.2 Hz, H-6), 3.88 (1H, s, -OCH_3), 3.71-3.87 (1H, m), 3.22-3.44 (1H, m), 2.87-2.93 (1H, m), 1.73 (1H, s, Ha); ^{13}C-NMR (100 MHz, $CDCl_3$) δ_C: 18.2 (q, C-b), 25.8 (q, C-a), 50.1 (d, C-8), 54.4 (d, C-8'), 55.9 (q, -OMe), 65.8 (t, C-e), 69.6 (t, C-9), 70.9 (t, C-9'), 82.0 (d, C-7), 87.6 (d, C-7'), 100.9 (t, O-CH_2-O), 106.4 (d, C-2), 108.1 (d, C-5), 109.3 (d, C-2'), 112.9 (d, C-5'), 118.4 (d, C-6'), 118.7 (d, C-6), 119.9 (d, C-d), 132.3 (s, C-1), 133.5 (s, C-1'), 137.5 (s, C-c), 146.5 (s, C-3), 147.6 (s, C-3'), 147.9 (s, C-4), 149.7 (s, C-4'); ESI-MS: *m/z* 425.0 $[M+H]^+$.

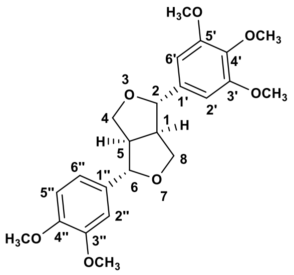

Compound **7**

(+)-magnolin **(7)**: $C_{23}H_{28}O_7$; ^{1}H-NMR (400 MHz, $CDCl_3$) δ_H: 7.13-6.65 (1H, m, Ar-H), 6.51 (1H, s, Ar-H), 4.71-4.69 (1H, m, H-2, H-6), 4.42-4.00 (1H, m, H-4, H-8), 4.08-3.61 (1H, m, H-4', H-8'), 3.81 (3H, s, -OMe), 3.77 (2H, s, -OMe), 3.37-2.73 (1H, m, H-1, H-5); ^{13}C-NMR (100 MHz, $CDCl_3$) δ_C: 53.0 (d, C-5), 53.4 (d, C-1), 54.9 (q, -OMe), 54.9 (q, -OMe), 55.1 (q, -OMe), 59.8 (q, -OMe), 70.7 (t, C-8), 70.9 (t, C-4), 84.7 (d, C-6), 85.0 (d, C-2), 101.7 (d, C-2'', C-6''), 108.2 (d, C-2'), 110.0 (s, C-5'), 117.2 (d, C-6'), 132.4 (s, C-1'), 135.8 (s, C-1''), 136.4 (s, C-4''), 147.6 (s, C-3'), 148.2 (s, C-4'), 152.4 (s, C-3'', C-5''); ESI-MS: *m/z* 417.0 $[M+H]^+$.

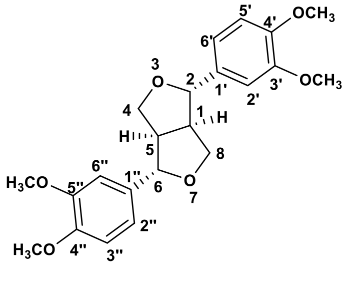

Compound **8**

(+)-Eudesmin **(8)**: $C_{22}H_{26}O_6$; ^{1}H-NMR (400 MHz, $CDCl_3$) δ_H: 7.26 (1H, s, H-8), 7.04-6.80 (1H, m,H-6'), 4.76 (2H, d, J = 4 Hz, H-4), 4.76 (2H, d, J = 4 Hz, H-2), 4.26 (2H, dd, J = 7.9 Hz, H-4'), 3.88, 3.90 (4 ×OMe), 3.10-3.13 (2H, m, H-1, H-5), 1.58(1H, s); ^{13}C-NMR (100 MHz, $CDCl_3$) δ_C: 54.1 (d, C-1, C-5), 55.9 (q, -OMe), 55.9

(q, -OMe), 71.7 (t, C-4, C-8), 85.8 (d, C-2, C-6), 109.2 (d, C-2', C-2''), 111.0 (s, C-5', C-5''), 118.2 (s, C-6', C-6''), 133.5 (s, C-1', C-1''), 148.6 (s, C-3', C-3''), 149.2 (s, C-4', C-4''); ESI-MS: *m/z* 387.0 $[M+H]^+$.

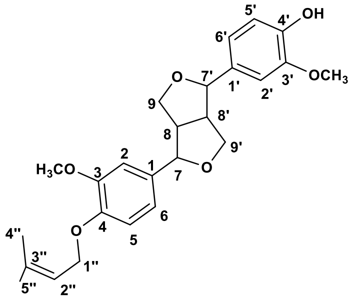

Compound **9**

***planisspine A* (9)**: $C_{25}H_{30}O_6$; ^{1}H-NMR (400 MHz, $CDCl_3$) δ_H: 7.26 (1H, s, H-3''), 6.92 (2H, d, J = 8.5 Hz, H-5), 6.90 (1H, s, H-6), 6.88 (1H, s, H-6'), 6.86 (2H, d, H-5', H-2), 6.81 (1H, d, J = 8.0 Hz, H-2'), 5.61 (1H, s, 4'-OH), 5.51 (1H, t, J = 6.0 Hz, H-2''), 4.86 (2H, d, J = 5.3 Hz, H-7), 4.58 (2H, d, J = 6.5 Hz, H-1''), 4.45 (2H, d, J = 7.0 Hz, H-8), 4.13 (1H, d, J = 7.0 Hz), 3.91 (3H, s, 3'-OCH_3), 3.88 (3H, s, 3-OCH_3), 3.87 (2H, t, J = 7.0 Hz, H-9β, H-9'β), 3.33 (2H, d, J = 5.0 Hz, H-8, H-9α), 2.92-2.81 (1H, m, H-8'); ^{13}C-NMR (100 MHz, $CDCl_3$) δ_C: 18.2 (q, C-5''), 25.8 (q, C-4''), 50.1 (d, C-8), 55.4 (d, C-8'), 55.9 (q, 3-OCH_3), 56.0 (q, 3'-OCH_3), 65.8 (t, C-1''), 69.7 (t, C-9'), 71.0 (t, C-9), 82.1 (d, C-7), 87.7 (d, C-7'), 108.3 (d, C-2), 109.3 (s, C-3''), 112.9 (d, C-2'), 114.2 (d, C-5'), 118.4 (d, C-5), 118.4 (d, C-6'), 119.9 (d, C-2''), 130.3 (d, C-6), 133.6 (s, C-1'), 137.6 (s, C-1), 144.6 (s, C-4'), 146.4 (s, C-3'), 147.9 (s, C-4), 149.7 (s, C-3); ESI-MS: *m/z* 427.1 $[M+H]^+$.

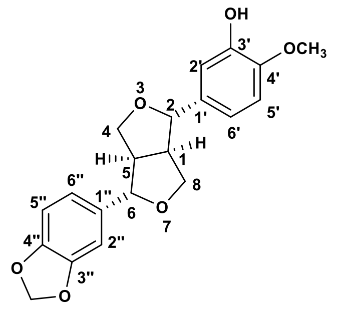

Compound **10**

***(+)-piperitol* (10)**: $C_{20}H_{20}O_6$; ^{1}H-NMR (400 MHz, $CDCl_3$) δ_H: 6.90-6.88 (2H, m, H-2', H-2''), 6.87-6.81 (2H, m, H-6', H-6''), 6.81-6.76 (2H, m, H-5', H-5''), 5.95 (2H, s, -OCH_2O), 5.60 (1H, s, -OH), 4.74-4.70 (2H, m, H-2, H-6), 4.45-4.09 (2H, m, H-4 e, H-8 e), 3.91 (3H, s, -OCH_3), 3.84-3.90 (2H, m, H-1, H-5), 3.33-2.66 (2H, m, H-1, H-5); ^{13}C-NMR (100 MHz, $CDCl_3$) δ_C: 54.1 (d, C-1), 54.3 (d, C-5), 55.9 (q, -OCH_3), 71.6 (t, C-4), 71.7 (t, C-8), 85.8 (d, C-2), 85.8 (d, C-6), 101.0 (t, -OCH_2O), 106.5 (d, C-2''), 108.2 (d, C-5''), 108.5 (d, C-2'), 114.2 (d, C-5'), 119.0 (d, C-6'), 119.3 (d, C-6''), 132.8 (s, C-1'), 135.1 (s, C-1''), 145.2 (s, C-4'), 146.7 (s, C-3''), 147.7 (s, C-3'), 147.9 (s, C-4''); ESI-MS: *m/z* 357.1 $[M+H]^+$.

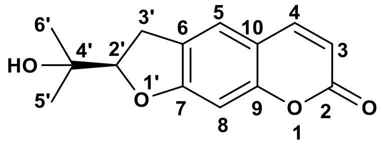

Compound **11**

***Nodakenetin* (11)**: $C_{14}H_{14}O_4$; ^{1}H-NMR (400 MHz, $CDCl_3$) δ_H: 7.59 (1H, d, J = 9.5 Hz, H-4), 7.27 (1H, s, H-5), 6.74 (1H, s, H-8), 6.21 (1H, d, J = 9.4 Hz, H-3), 4.73 (1H, t, J = 8.5 Hz, H-2'), 3.22 (2H, m, H-3'), 1.37 (3H, s, CH_3), 1.24 (3H, s, CH_3); ^{13}C-NMR (100 MHz, $CDCl_3$) δ_C: 24.2 (q, C-5'), 26.1 (q, C-6'), 29.5 (t, C-3'), 71.6 (s, C-4'), 91.1

(d, C-2'), 97.9 (d, C-8), 112.3 (d, C-3), 112.7 (s, C-9), 123.4 (d, C-5), 125.0 (s, C-6), 143.6 (d, C-4), 155.6 (s, C-10), 161.4 (s, C-7), 163.1 (s, C-2); ESI-MS: *m/z* 247.0 $[M+H]^+$.

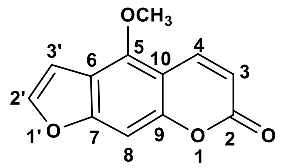

Compound **12**

***Bergapten* (12)**: $C_{12}H_8O_4$; ^{1}H-NMR (400 MHz, $CDCl_3$) δ_H: 8.15 (1H, d, *J* = 9.8 Hz, H-4), 7.59 (1H, d, *J* = 2.4 Hz, H-2'), 7.26 (1H, s, H-8), 7.02 (1H, d, *J* = 2.4 Hz, H-3'), 6.27 (1H, d, *J* = 9.8 Hz, H-3), 4.27(3H, s, 5-OCH_3); ^{13}C-NMR (100 MHz, $CDCl_3$) δ_C: 60.1 (q, 5-OCH_3), 93.8 (d, C-8), 105.0 (d, C-3'), 106.4 (s, C-10), 112.5 (s, C-3), 112.6 (s, C-6), 139.2 (d, C-4), 144.8 (d, C-2'), 149.5 (s, C-5), 152.7 (sC-, 9), 158.4 (s, C-7), 161.2 (s, C-2); ESI-MS: *m/z* 217.1 $[M+H]^+$.

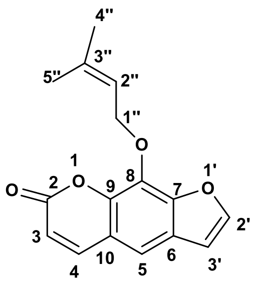

Compound **13**

***Imperatorin* (13)**: $C_{16}H_{14}O_4$; ^{1}H-NMR (400 MHz, $CDCl_3$) δ_H: 7.76 (1H, d, *J* = 9.6 Hz, H-4), 7.69 (1H, d, *J* = 2.3 Hz, H-2'), 7.36 (1H, s, H-5), 6.81 (1H, d, *J* = 2.2 Hz, H-3'), 6.36 (1H, d, *J* = 9.6 Hz, H-3), 5.61 (1H, t, *J* = 7.3 Hz, H-2''), 5.00 (2H, d, *J* = 7.2 Hz,

H-1''), 1.74 (3H, s, H-4''), 1.72 (3H, s, H-3''); ^{13}C-NMR (100 MHz, $CDCl_3$) δ_C: 18.1 (q, C-5''), 25.8 (q, C-4''), 70.1 (t, C-1''), 106.7 (d, C-3'), 113.1 (d, C-5), 114.6 (d, C-3), 116.4 (s, C-10), 119.7 (d, C-2''), 125.8 (s, C-6), 131.6 (s, C-8), 139.7 (s, C-3''), 143.8 (s, C-9), 144.3 (d, C-4), 146.6 (d, C-2'), 148.6 (d, C-7), 160.5 (s, C-2); ESI-MS: *m/z* 271.1 $[M+H]^+$.

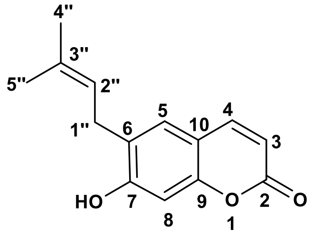

Compound **14**

Demethylsuberosin **(14)**: $C_{14}H_{14}O_3$; ^{1}H-NMR (400 MHz, $CDCl_3$) δ_H: 7.63 (1H, d, J = 9.4 Hz, H-4), 7.26 (3H, s, H-5), 7.19 (1H, s, H-8), 6.23 (1H, d, J = 9.5 Hz, H-3), 5.31 (1H, t, J = 6.8 Hz), 3.38 (2H, d, J = 6.8 Hz), 1.80 (3 H, s), 1.74 (3 H, s); ^{13}C-NMR (100 MHz, $CDCl_3$) δ_C: 17.9 (q, C-5''), 25.8 (q, C-4''), 28.7 (t, C-1''), 103.3 (s, C-3''), 112.4 (d, C-5), 112.6 (d, C-3), 120.9 (s, C-10), 125.2 (d, C-2''), 128.3 (s, C-6), 135.4 (d, C-8), 143.8 (s, C-9), 158.2 (s, C-7), 161.9 (s, C-2); ESI-MS: *m/z* 231.0 $[M+H]^+$.

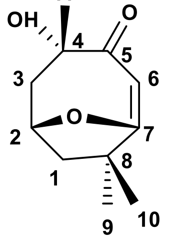

Compound **15**

Pubinernoid A **(15)**: $C_{11}H_{16}O_3$; ^{1}H-NMR (400 MHz, $CDCl_3$) δ_H: 2.29 (1H, d, H-3),

1.79 (3H, s), 1.47 (3H, s, H-10), 1.27 (3H, s, H-9), 1.20 (2H, s, H-2), 0.98 (1H, s, H-6); ^{13}C-NMR (100 MHz, $CDCl_3$) δ_C: 26.4 (q, C-9), 26.9 (q, C-10), 30.6 (q, C-11), 35.9 (s, C-8), 45.6 (t, C-1), 47.2 (t, C-3), 66.7 (d, C-2), 86.7 (s, C-4), 112.8 (s, C-7), 172.1 (d, C-6), 182.7 (s, C-5); ESI-MS: *m/z* 197.1[M+H]$^+$.

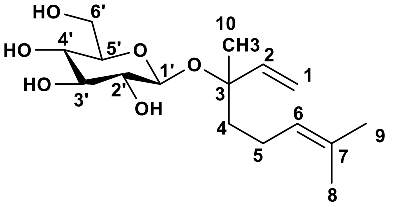

Compound **16**

Linalyl-β-glucopyranoside **(16)**: $C_{16}H_{28}O_6$; ^{1}H-NMR (400 MHz, $CDCl_3$) δ_H: 7.20 (1H, s), 5.90 (1H, dd, *J* = 17.3, 11.4 Hz, H-2), 5.10 (1H, d, *J* = 18.0 Hz, H-1-b), 5.07 (1H, d, *J* = 11.0 Hz, H-1-a), 5.12 (1H, t, *J* = 7.0 Hz, H-6), 4.27 (3H, d, *J* = 7.7 Hz, H-1'), 3.70 (6H, dd, *J* = 11.8, 2.2 Hz, H-6'-a), 3.46 (6H, dd, *J* = 11.8, 5.4 Hz, H-6'-b), 3.44 (2H, m, H-4'), 3.31 (2H, m, H-3'), 3.12 (1H, m, H-2'), 1.91 (2H, m, H-5), 1.60 (3H, s, H-9), 1.59 (2H, m, H-5), 1.51 (3H, s, H-8), 1.24 (3H, s, H-10); ^{13}C-NMR (100 MHz, $CDCl_3$) δ_C: 16.7 (q, C-8), 21.6 (q, C-10), 21.6 (t, C-5), 24.6 (q, C-9), 38.9 (t, C-4), 60.7 (t, C-6'), 68.5 (d, C-4'), 72.1 (d, C-2'), 74.2 (d, C-5'), 79.7 (s, C-3), 79.7 (d, C-3'), 96.5 (q, C-1), 113.8 (d, C-1'), 123.3 (d, C-6), 130.6 (s, C-7), 141.7 (d, C-2); ESI-MS: *m/z* 339.0 [M+Na]$^+$.

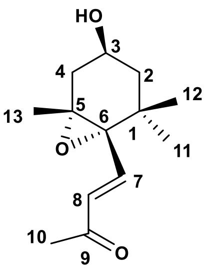

Compound **17**

(3S,5R,6S,7E)-5,6-epoxy-3-hydroxy-7-megastigmen-9-one **(17)**: $C_{13}H_{20}O_3$; ^{1}H-NMR (400 MHz, $CDCl_3$) δ_H: 6.96 (1H, d, J = 15.6 Hz, H-6), 6.22 (1H, d, J = 15.6 Hz, H-7), 3.81 (1H, m, H-3), 2.33 (1H, dd, J = 9.2, 5.2 Hz, H-4), 2.21 (3H, s, H-10), 1.60 (1H, dd, J = 11.5, 9.2 Hz, H-4), 1.56 (1H, dd, J = 12.2, 4.5 Hz, H-2), 1.40 (1H, s, H-13), 1.2 (1H, dd, J = 12.2, 10.2 Hz, H-1), 1.13 (3H, s, H-11), 0.91 (3H, s, H-12); ^{13}C-NMR (100 MHz, $CDCl_3$) δ_C: 18.8 (q, C-13), 23.9 (q, C-12), 27.2 (q, C-11), 28.3 (q, C-10), 34.0 (s, C-1), 39.5 (t, C-2), 45.6 (t, C-4), 63.0 (d, C-3), 66.2 (s, C-5), 68.4 (s, C-6), 131.5 (d, C-8), 141.4 (d, C-7), 196.4 (s, C-9); ESI-MS: *m/z* 225.1 $[M+H]^+$.

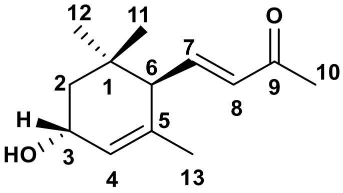

Compound **18**

(3R,6R,7E)-3-hydroxy-4,7-megastigmadien-9-one **(18)**: $C_{13}H_{20}O_2$; ^{1}H-NMR (400 MHz, $CDCl_3$) δ_H: 6.89 (1H, dd, J = 15.8, 10.0 Hz, H-7), 6.55 (1H, d, J = 15.9 Hz, H-8), 5.5 (1H, s, H-4), 3.89 (3H, s, H-3), 2.12 (1H, d, J = 10.0 Hz, H-6), 2.08 (1H, s, H-10), 1.70 (2H, dd, J = 13.4, 5.8 Hz, H-2), 1.60 (3H, s, H-13), 1.29 (1H, dd, J = 13.4, 6.4 Hz, H-2), 1.01 (1H, s, H-11), 0.63 (1H, s, H-12); ^{13}C-NMR (100 MHz, $CDCl_3$) δ_C:

22.6 (q, C-13), 24.6 (q, C-12), 27.2 (q, C-10), 29.3 (q, C-11), 33.8 (s, C-1), 43.8 (t, C-2), 54.2 (d, C-6), 65.4 (d, C-3), 125.8 (d, C-4), 133.6 (d, C-8), 135.4 (s, C-5), 147.1 (d, C-7), 198.1 (s, C-9); ESI-MS: *m/z* 209.0 $[M+H]^+$.

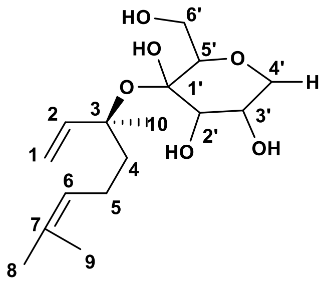

Compound **19**

Linalyl-O-β-D-glucoside **(19)**: $C_{16}H_{28}O_6$; ^{1}H-NMR (400 MHz, $CDCl_3$) δ_H: 5.97 (1 H, dd, *J* = 17.3, 11.4 Hz, H-2), 5.21 (1H, dd, *J* = 17.6, 1.3 Hz, H-1b), 5.17 (1H, dd, *J* = 11.0, 1.3 Hz, H-1a), 5.08 (1H, m, H-6), 4.34 (1H, d, *J* = 7.7 Hz, H-1'), 3.77 (2H, m, H-6'), 3.66-3.29 (4H, m, H-2', H-5'), 1.98 (2H, m, H-5), 1.67 (3H, s, H-9), 1.64 (2H, m, H-4), 1.34 (3H, s, H-8), 1.31 (3H, s, H-10); ^{13}C-NMR (100 MHz, $CDCl_3$) δ_C: 17.7 (q, C-8), 22.6 (t, C-5), 22.6 (q, C-9), 25.6 (q, C-10), 39.9 (t, C-4), 61.6 (t, C-6'), 69.4 (d, C-4'), 73.1 (d, C-2'), 75.3 (s, C-3), 80.8 (d, C-5'), 97.5 (s, C-3'), 114.9 (t, C-1), 124.3 (d, C-6), 131.6 (s, C-7), 142.7 (d, C-2); ESI-MS: *m/z* 339.1 $[M+Na]^+$.

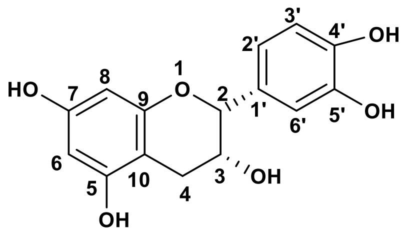

Compound **20**

(-) epicatechin **(20)**: $C_{15}H_{14}O_6$; ^{1}H-NMR (400 MHz, DMSO) δ_H: 6.89 (1H, s, H-2'),

6.66 (1H, s, H-5'), 6.66 (1H, s, H-6'), 5.89 (1H, d, J = 2.3 Hz, H-8), 5.72 (1H, d, J = 2.3 Hz, H-6), 4.74 (1H, s, H-2), 4.16 (1H, s, H-3), 2.66 (1H, dd, J = 4.6,16.6 Hz, H-4), 2.51 (1H, dd, J = 3.4, 16.6 Hz, H-4); ^{13}C-NMR (100 MHz, MeOH) δ_C: 27.8 (t, C-4), 66.1 (d, C-3), 78.4 (d, C-2), 94.5 (d, C-8), 95.0 (d, C-6), 98.7 (s, C-10), 113.9 (d, C-2'), 114.5 (d, C-6'), 118.0 (s, C-5'), 130.9 (s, C-1'), 144.3 (s, C-3'), 144.5 (s, C-4'), 155.9 (s, C-9), 156.2 (s, C-5), 156.6 (s, C-7); ESI-MS: *m/z* 291.0 $[M+H]^+$.

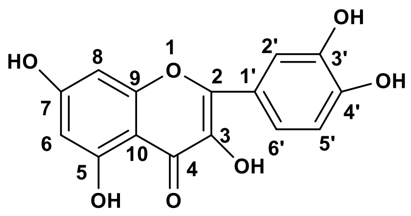

Compound **21**

***Quercetin* (21)**: $C_{15}H_{10}O_7$; ^{1}H-NMR (400 MHz, DMSO) δ_H: 7.68 (1H, d, J = 2.2 Hz, H-2'), 7.54 (1H, dd, J = 2.2 Hz, 8.5 Hz, H-6'), 6.89 (1H, d, J = 8.2 Hz, H-5'), 6.41 (1H, d, J = 2.0 Hz, H-6), 6.19 (1H, d, J = 2.0 Hz, H-8); ^{13}C-NMR (100 MHz, MeOH) δ_C: 93.8 (d, C-8), 98.6 (d, C-6), 103.4 (s, C-10), 115.0 (d, C-2'), 116.0 (d, C-6'), 120.4 (d, C-5'), 122.4 (s, C-1'), 136.9 (s, C-3), 144.5 (s, C-3'), 147.2 (s, C-2), 148.1 (s, C-4'), 156.5 (s, C-9), 161.1 (s, C-5), 164.3 (s, C-7), 176.2 (s, C-4); ESI-MS: *m/z* 303.0 $[M+H]^+$.

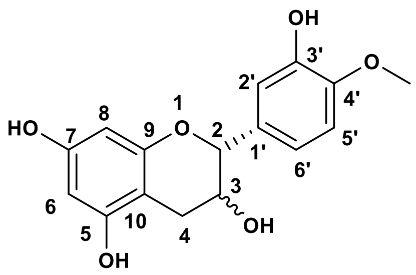

Compound **22**

4'-O-methyl catechin **(22)**: $C_{16}H_{16}O_6$; ^{1}H-NMR (400 MHz, MeOH) δ_H: 7.14 (1H, s, OH-7), 7,12 (1H, s, OH-3'), 6.91 (1H, d, J = 1.9 Hz, H-5'), 6.89 (1H, d, J = 1.9 Hz, H-2'), 6.78 (1H, dd, J = 2.0, 8.3 Hz, H-6'), 5.93 (1H, dd, J = 12.0, 2.2 Hz, H-6, H-8), 4.85 (1H, d, J = 6.5 Hz, OH-3), 3.86 (3H, s, H-2), 3.34 (1H, s, OCH_3), 3.31 (1H, dddd, J = 5.4, 6.5, 8.0, 8.2 Hz, H-3), 2.88 (1H, dd, J = 16.8 Hz, 4.6 Hz, H-4α), 2.75 (1H, dd, J = 16.9 Hz, 2.9 Hz, H-4β); ^{13}C-NMR (100 MHz, MeOH) δ_C: 28.0 (t, C-4), 54.9 (q, OCH_3), 66.2 (d, C-3), 78.6 (d, C-2), 94.5 (d, C-8), 95.0 (d, C-6), 98.6 (s, C-10), 110.4 (d, C-5'), 114.2 (d, C-2'), 119.2 (d, C-6'), 130.9 (s, C-1'), 145.6 (s, C-3'), 147.2 (s, C-4'), 155.9 (s, C-9), 156.3 (s, C-5), 156.6 (s, C-7); ESI-MS: m/z 305.1 $[M+H]^+$.

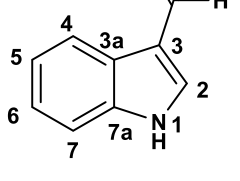

Compound **23**

1H-indole-3-carbaldehyde **(23)**: C_9H_7NO; ^{1}H-NMR (400 MHz, MeOH) δ_H: 9.88 (1H, s, 3-CHO), 8.26 (1H, d, J = 2.0 Hz, H-2), 8.09 (1H, d, J = 7.6 Hz, H-4), 7.49 (1H, d, J = 8.0 Hz, H-7), 7.28 (1H, dd, J = 8.0, 7.8 Hz, H-6), 7.24 (1H, dd, J = 7.8, 7.6 Hz, H-5); ^{13}C-NMR (100 MHz, MeOH) δ_C: 112.8 (d, C-7), 118.6 (s, C-3), 121.2 (s, C-4), 122.5

(d, C-5), 123.9 (d, C-6), 124.5 (s, C-3a), 137.5 (s, C-7a), 138.9 (d, C-2), 185.4 (q, 3-CHO); ESI-MS: *m/z* 146.1 $[M+H]^+$.

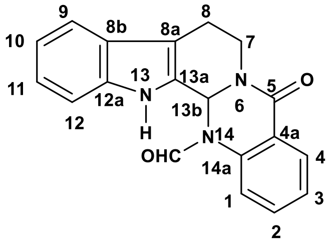

Compound **24**

N^14^-formyldihydrorutaecarpine **(24)**: $C_{19}H_{15}N_3O_2$; ^{1}H-NMR (400 MHz, DMSO) δ_H: 11.14 (1H, s, N-H), 9.12 (1H, s, -CHO), 7.88 (1H, t, *J* = 7.5 Hz, H-2), 7.63 (1H, d, *J* = 8.0 Hz, H-9), 7.58 (1H, d, *J* = 8.0 Hz, H-9), 7.37 (1H, d, *J* = 7.9 Hz, H-12), 7.30 (1H, t, *J* = 8.0 Hz, H-11), 7.26 (1H, d, *J* = 8.2 Hz, H-1), 7.05 (1H, t, *J* = 7.6 Hz, H-10), 6.96 (1H, t, *J* = 7.5 Hz, H-3), 4.68 (1H, dd, *J* = 12.5, 5.0 Hz, Ha-7), 3.62 (1H, td, *J* = 12.5, 5.0 Hz, Hb-7), 3.01 (1H, dddd, *J* = 15.8, 11.5, 6.2, 2.3 Hz, Ha-8), 2.65 (1H, dd, *J* = 16.0, 4.8 Hz, Hb-8); ^{13}C-NMR (100 MHz, DMSO) δ_C: 19.5 (t, C-8), 44.0 (t, C-7), 62.2 (s, C-13b), 111.1 (d, C-12), 118.3 (d, C-1), 118.5 (d, C-9), 119.4 (d, C-10), 122.2 (d, C-11), 125.8 (s, C-8b), 128.6 (s, C-4a), 131.4 (s, C-13a), 134.4 (d, C-2), 136.1 (s, C-12a), 137.4 (s, C-14a), 162.5 (d, N-CHO), 163.6 (s, C-5); ESI-MS: *m/z* 318.1 $[M+H]^+$.

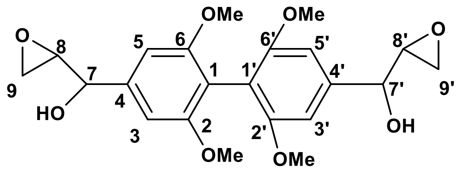

Compound **25**

2,6,2',6'-tetramethoxy-4,4'-bis(2,3-epoxy-1-hydroxypropyl) biphenyl **(25)**: $C_{22}H_{26}O_8$; ^{1}H-NMR (400 MHz, $CDCl_3$) δ_H: 6.59 (4H, s, H-3, H-3', H-5, H-5'), 5.60 (2H, s, OH-7, OH-7'), 4.74 (2H, d, J = 4.1 Hz, H-7, H-7'), 4.26 (2H, m, H-9 b, H-9 b'), 3.91 (2H, m, H-9 a, H-9 a'), 3.89 (12H, s, OMe-2, OMe-2', OMe-6, OMe-6'), 3.09 (2H, m, H-8, H-8'); ^{13}C-NMR (100 MHz, $CDCl_3$) δ_C: 54.5 (d, C-8, C-8'), 56.6 (q, OMe-3, 3' ,5 ,5'), 72.0 (t, C-9, C-9'), 86.3 (d, C-7, C-7'), 102.9 (d, C-3, C-3', C-5, C-5'), 132.3 (s, C-1, C-1'), 134.5 (s, C-4, C-4'), 147.3 (s, C-2, C-2', C-6, C-6'); ESI-MS: *m/z* 419.1 $[M+H]^+$.

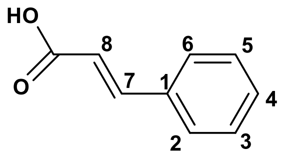

Compound **26**

cinnamic acid **(26)**: $C_9H_8O_2$; ^{1}H-NMR (400 MHz, $CDCl_3$) δ_H: 7.80 (1H, d, J = 16.0 Hz, H-8), 7.54 (2H, dd, J = 5.6, 2.0 Hz, H-6, H-2), 7.41 (3H, m, H-3, H-4, H-5), 6.47 (1H, d, J = 16.0 Hz, H-7); ^{13}C-NMR (100 MHz, $CDCl_3$) δ_C: 117.2 (d, C-8), 128.3 (d, C-3, C-5), 128.9 (d, C-2, C-6), 130.7 (d, C-4), 134.0 (s, C-1), 147.0 (s, -COOH), 172.1 (d, C-7); ESI-MS: *m/z* 149.1 $[M+H]^+$.

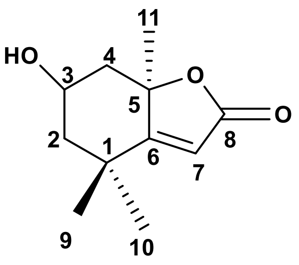

Compound **27**

***Loliolide* (27)**: $C_{11}H_{16}O_3$; ^{1}H-NMR (400 MHz, $CDCl_3$) δ_H: 5.69 (1H, s, H-7), 4.34 (1H, m, H-3), 2.47 (1H, dd, J = 14.1, 3.6 Hz, H-4a), 1.97 (1H, dd, J = 14.1, 3.6 Hz, H-2a), 1.79 (3H, s, H-11), 1.66 (1H, dd, J = 14.4, 3.6 Hz, H-4b), 1.54 (1H, dd, J = 14.4, 3.6 Hz, H-2b), 1.47 (3H, s, H-10), 1.27 (3H, s, H-9); ^{13}C-NMR (100 MHz, $CDCl_3$) δ_C: 26.4 (q, C-11), 26.9 (q, C-10), 30.6 (q, C-9), 35.9 (s, C-1), 45.6 (t, C-4), 47.2 (t, C-2), 66.7 (d, C-3), 86.8 (s, C-5), 112.8 (d, C-7), 172.0 (s, C-8), 182.5 (s, C-6); ESI-MS: *m/z* 197.1 $[M+H]^+$.

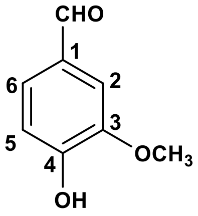

Compound **28**

***4-hydroxy-3-methoxy-benzaldehyde* (28)**: $C_8H_8O_3$; ^{1}H-NMR (400 MHz, $CDCl_3$) δ_H: 9.83 (1H, s, CHO-1), 7.43 (1H, dd, J = 8.5, 1.6 Hz, H-6), 7.42 (1H, d, J = 1.4 Hz, H-2), 7.05 (1H, d, J = 8.5 Hz, H-5), 6.24 (1H, s, HO-4), 3.96 (3H, s, CH_3O-3); ^{13}C-NMR (100 MHz, $CDCl_3$) δ_C: 56.1 (q, CH_3O-3), 108.7 (d, C-5), 114.3 (d, C-2), 127.5 (d, C-6), 129.9 (s, C-1), 147.1 (s, C-3), 151.6 (s, C-4), 190.9 (d, CHO-1); ESI-MS: *m/z* 175.1 $[M+Na]^+$.

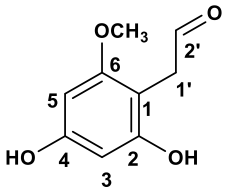

Compound **29**

***2,4-dihydroxy-6-methoxy acetophenone* (29)**: $C_9H_{10}O_4$; ^{1}H-NMR (400 MHz, DMSO) δ_H: 2.52 (3H, s, H-1'), 3.82 (3H, s, -OMe), 5.87 (1H, d, J = 2.2 Hz, H-5), 5.97 (1H, d, J = 2.2 Hz, H-3), 10.65 (1H, s, H-2'), 13.8 (1H, s, OH-2); ^{13}C-NMR (100 MHz, DMSO) δ_C: 33.0 (t, C-1'), 56.2 (q, -OCH_3), 91.7 (d, C-5), 96.0 (d, C-3), 105.0 (s, C-1), 163.8 (s, C-4), 165.5 (s, C-6), 166.7 (s, C-2), 202.7 (s, C-2'); ESI-MS: *m/z* 183.1 $[M+H]^+$.

2.Separation methods of the compounds

Fraction Y-7 (29.5 g) was separated by a RP-C 18 column, eluting with methanol /water (from 30 to 100%, 2 L for each ratio), to afford twelve subfractions (fractions Y-7-1 to Y-7-12). Fractions Y-7-1 (2.24 g), Y-7-2 (542.2 mg), Y-7-3 (283 mg), Y-7-4 (619.3 mg), Y-7-6 (707 mg) and Y-7-7 (783 mg) were purified by Sephadex LH-20 column chromatography to afford compound **20** (30 mg), compound **25** (99 mg), compound **21** (22 mg), and compound **16** (22 mg). Compound **1** (5.8 mg) and compound **2** (12 mg) from fraction Y-7-6-1 (63 mg) were obtained by silica gel column chromatography eluting with chloroform/acetone (from 7:1 to 4:1). A silica

gel column was eluted with chloroform/methanol (7:1) and Y-7-3 (99 mg) to yield compound **22** (6.8 mg), Y-7-7-2 (168.1 mg) to yield compound **19** (30 mg).

Fraction Y-6 (51 g) was subjected over a silica gel column (200-300 mesh, 0.8 kg) chromatography eluted with petroleum ether/ethyl acetate (from 10:1 to 1:1, 6 L for each ratio) to give ten fractions (fractions Y-6-1 to Y-6-10), the fraction Y-6-5 (2.388 g), Y-6-8 (4.59 g), Y-6-9 (3 g), Y-6-10 (4.24 g) separated by a RP-C 18 column, eluting with methanol/water (from 30 to 100%, 2 L for each ratio), fraction Y-6-5-3 (14.8 mg), Y-6-8-1 (55 mg), Y-6-9-1 (88 mg), Y-6-9-2 (34 mg), Y-6-9-3 (56 mg), Y-6-10-1 (76 mg), Y-6-10-2 (24 mg), Y-6-10-5 (40 mg) were subjected over a silica gel column chromatography eluted with chloroform/methanol (from 1:0 to 10:1, 300 mL for each ratio) to afford compound **23** (4.5 mg), compound **15** (16 mg), compound **17** (5.7 mg), compound **26** (6.5 mg), compound **11** (5.5 mg), compound **7** (4.7 mg), compound **8** (5.5 mg), compound **24** (7 mg), compound **27** (9 mg).

Fraction Y-5 (40 g) was separated by a RP-C 18 column, eluting with methanol/water (from 30 to 100%, 2 L for each ratio), to afford ten subfractions (fractions Y-5-1 to Y-5-10). Fractions Y-5-2 (174.7 mg), Y-5-3 (178 mg), Y-5-4 (102.7 mg), Y-5-6 (430 mg), Y-5-8 (382.9 mg) and Y-5-9 (65 mg) were eluted by silica gel column chromatography with chloroform/methanol (1:0 to 10:1) to afford compound **28** (9 mg), compound **18** (7 mg), compound **3** (5 mg), compound **5** (27 mg), compound **12** (7 mg), compound **13** (16.1 mg), compound **4** (34 mg), and compound **6** (20 mg).

Fraction Y-4 (27 g) was separated by a RP-C 18 column, and eluted with

methanol/water (from 50 to 100%, 2 L for each ratio) to afford nine subfractions (fractions Y-4-1 to Y-4-9). Fraction Y-4-3 (43 mg) was subjected to silica gel column chromatography with petroleum ether/ethyl acetate (7:1 to 1:1) to afford compound **29** (7 mg). Fraction Y-4-4 (43 mg) was purified by semipreparative HPLC (50:50 acetonitrile/water, 2 mL/min) to yield compound **10** (6 mg, t_R =9 min), compound **14** (3.3 mg, t_R =15 min), and compound **9** (25 mg, t_R =20 min)

3.Determination of MICs and MFCs

Table S1. Antifungal bioactivity of 29 compounds

Strain	Compounds	MICs (μg/mL)	MFCs(μg/mL)
	**1**	64	> 128
	**2**	32	> 128
	**3**	32	> 128
	**9**	64	> 128
	**10**	32	> 128
	**11**	64	> 128
C. albicans (08030401)	**20**	64	> 128
	**21**	64	> 128
	**25**	32	> 128
	**26**	32	> 128
	**28**	32	> 128
	**29**	32	> 128

Schematic illustration of the checkerboard assay

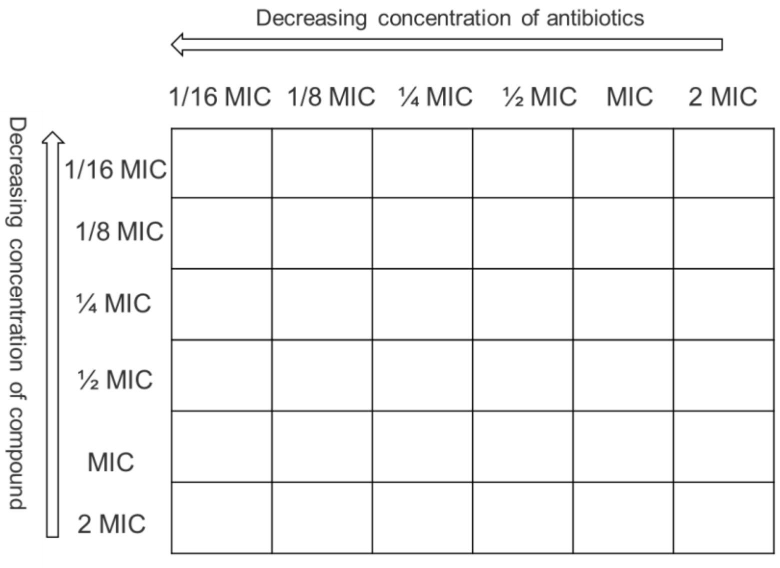

Fig. S1 Schematic illustration of the checkerboard assay.

X = Fluconazole, Y = compounds **3**, **10** or **29.**

4.Antibiofilm activity and SEM.

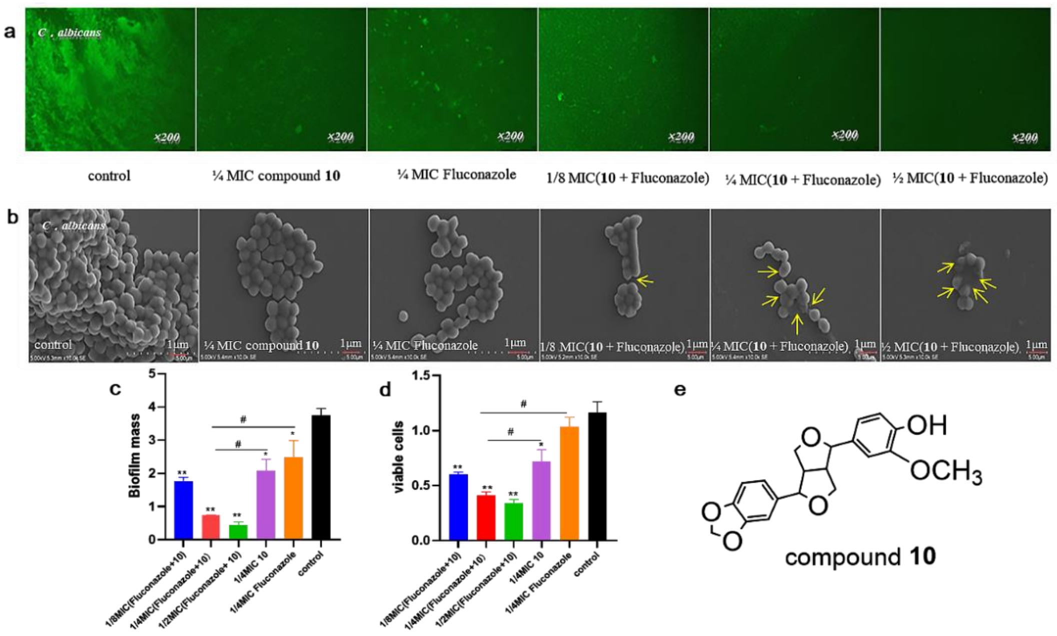

Fig. S2 *C. albicans* were treated with compound **10** and Fluconazole alone or in combination. (a) Biofilms were observed under a fluorescence microscope at 200×. (b)

SEM photography of *C. albicans*. The yellow arrows represent the observed morphological changes. (c) Biofilms were detected by the MTT method. (d) Biofilms were detected by the crystal violet method. (e) The chemical structure of compound. Values were mean ± SD. $^{*}P < 0.05$, $^{**}P < 0.01$, $^{***}P < 0.001$ versus control. $^{\#}P < 0.05$, $^{\#\#}P < 0.01$ versus ¼ Fluconazole or ¼ compound.

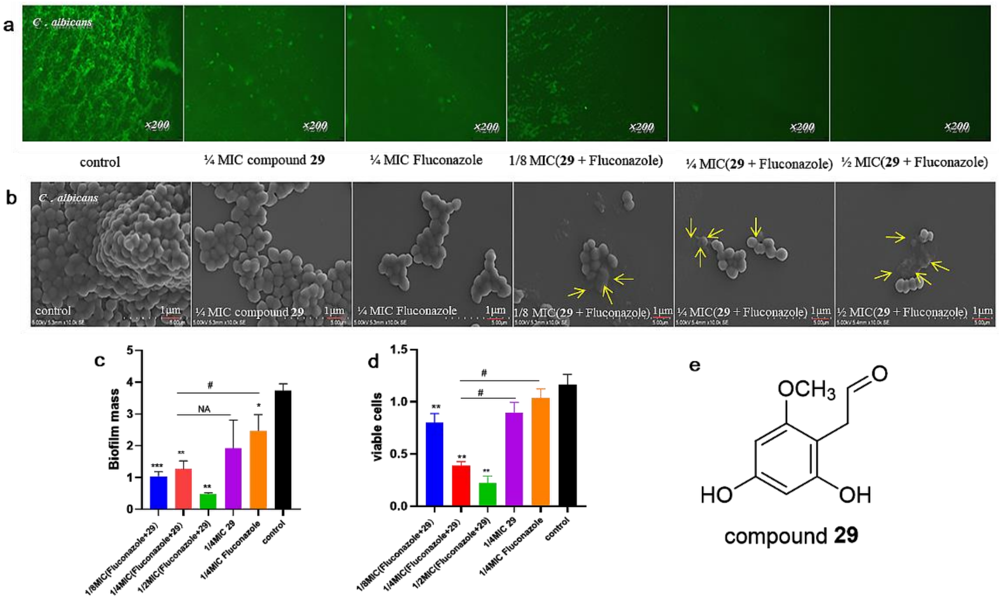

Fig. S3 *C. albicans* were treated with compound **29** and Fluconazole alone or in combination. (a) Biofilms were observed under a fluorescence microscope at 200×. (b) SEM photography of *C. albicans*. The yellow arrows represent the observed morphological changes. (c) Biofilms were detected by the MTT method. (d) Biofilms were detected by the crystal violet method. (e) The chemical structure of compound. Values were mean ± SD. $^{*}P < 0.05$, $^{**}P < 0.01$, $^{***}P < 0.001$ versus control. $^{\#}P < 0.05$, $^{\#\#}P < 0.01$ versus ¼ Fluconazole or ¼ compound.

5.Cytotoxic assay

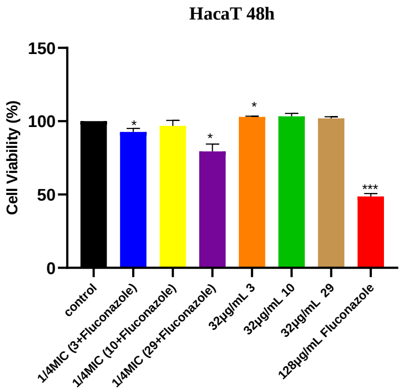

Fig. S4 The 1/ 4 MIC of the compounds (**3**, **10**, **29**) combined with Fluconazole, the MIC of the compounds (**3**, **10**, **29**), and the MIC of Fluconazole were tested for cytotoxic activity against HaCaT cells *in vitro* by the MTT method. Values were the mean ± SD.$^{*}P < 0.05$, $^{**}P < 0.01$, $^{***}P < 0.001$ versus control.

6. S1. References

Anna, V.-Q., Arnaud, V., Emmanuelle, M., Veronique, C., & Nicolas, S. (2015). Straightforward Method To Quantify GSH, GSSG, GRP, and Hydroxycinnamic Acids in Wines by UPLC-MRM-MS. *Journal of Agricultural and Food Chemistry, 63*, 142-149.

Chen, B. R., Liu, Q., Wang, H., Gao, Z. Y., Siddeeg, A., & Zhu.S.M. (2020). Purification, characterization, and identification of 3-hydroxy-4-methoxy benzal acrolein–an intermediate of synthesizing advantame. *Food Science &*

Nutrition, 8(2), 744-753.

Fei, D. Q., Arfan, M., Rafiq, J., & Gao, K. (2009). Chemical constituents from the aerial parts of Sophora mollis. *Chemistry of Natural Compounds, 45*, 896-897.

Gregory, B., Andrew, F. P., & Thomas, C. B. (2001). Manganese(III) acetate mediated radical reactions leading to araliopsine and related quinoline alkaloids. *Tetrahedron, 57*, 4719-4728.

Hoi-Seon, L. (2016). Insecticidal Toxicities and Essential Oil Compositions of *Zanthoxylum* piperitum and *Zanthoxylum* schinifolium Fruits in Korea. *Journal of Essential Oil Bearing Plants, 19*(8), 2065-2071.

Ke, J. X., Cheng, J. X., Luo, Q. Y., Wu, H. J., Shen, G. H., & Zhang, Z. Q. (2020). Identification of two bitter components in *Zanthoxylum bungeanum* Maxim. and exploration of their bitter taste mechanism through receptor hTAS2R14. *Food Chemistry, 338*, 127816.

Kumar, V., Kumar, S., Singh, B., & Kumar, N. (2014). Quantitative and structural analysis of amides and lignans in *Zanthoxylum* armatum by UPLC-DAD-ESI-QTOF-MS/MS. *Journal of Pharmaceutical and Biomedical Analysis, 94*(3), 23-29.

Luo, Z., Yu, L., Liu, Q., Chen, M., Chen, S., Chen, Y., & Wang, P. (2003). Efficient separation of α-pinene from turpentine. *Guangdong Chemical Industry, 30*(4), 4.

Masuda, T., Takasugi, M., & Anetai, M. (1998). Psoralen and other linear furanocoumarins as phytoalexins in Glehnia littoralis. *Phytochemistry, 47*(1),

13-16.

Noha, K., Mostafa, F., Mokhtar, B., Soheir, E.-Z., & Osama, S. (2017). Foliar spraying of salicylic acid induced accumulation of phenolics, increased radical scavenging activity and modified the composition of the essential oil of water stressed Thymus vulgaris L. *Plant Physiology and Biochemistry, 123*, 65-74.

Roja, S., Rajiv Kumar, K., Priyashree, S., Pritha, B., Puja, K., Salona, B., Sharad, S., & Shakti, P. P. (2020). LC-MS characterized methanolic extract of zanthoxylum armatum possess anti-breast cancer activity through Nrf2-Keap1 pathway: An in-silico, *in-vitro* and *in-vivo* evaluation. *Journal of Ethnopharmacology, 269*, 113758.

Saeed, M. A., & Sabir, A. W. (2007). Irritant and cytotoxic coumarins from Angelica glauca Edgew roots. *Journal of Asian Natural Products Research, 10*, 49-58.

Choi S. U., Yang M.C., Lee K. H., Kim K. H., Lee K. R. (2007). Lignan and terpene constituents from the aerial parts of saussurea pulchella. *Archives of Pharmacal Research, 30*, 1067-1074.

Takahiro, M., Mitsuo, T., & Masaki, A. (1998). Psoralen and other linear furanocoumarins as phytoalexins in Glehnia littoralis. *Phytochemistry, 47*, 13-16.

Vinod, B., Sushila, S., Neeraj, K., Upendra, S., & Bikram, S. (2016). Simultaneous quantification and identification of flavonoids, lignans, coumarin and amides in leaves of *Zanthoxylum* armatum using UPLC-DAD-ESI-QTOF–MS/MS. *Journal of Pharmaceutical and Biomedical Analysis, 132*, 46-55.

Vishal, K., Shiv, K., Bikram, S., & Neeraj, K. (2014). Quantitative and structural analysis of amides and lignans in Zanthoxylum armatum by UPLC-DAD-ESI-QTOF–MS/MS. *Journal of Pharmaceutical and Biomedical Analysis, 94*, 23-29.

Wang, C., Yang, Y., Mei, Z. N., & Yang, X. Z. (2013). Cytotoxic compounds from Laminaria japonica. *Chemistry of Natural Compounds, 49*, 699-701.

Wang, Z.-J., Zhou, Y., Shi, X.-L., Xiao, X., He, Y.-J., Zhu, Y.-Y., Xie, T.-Z., Liu, T., Xu, X.-J., & Luo, X.-D. (2021). Comparison of chemical constituents in diverse *zanthoxylum* herbs, and evaluation of their relative antibacterial and nematicidal activity. *Food Bioscience, 42*, 101206.

Yang, X. W., Zhang, H., & Jun, H. U. (2008). Chemical Constituents of Near Ripe Fruits of Evodia rutaecarpa var. bodinieri. *Journal of Tropical Subtropical Botany*(03), 60-64.

Yoro, T., Franck, R., Jean, C., Alassane, W., & Julien, P. (2017). A Method for LC-MS/MS Profiling of Coumarins in *Zanthoxylum zanthoxyloides* (Lam.) B. Zepernich and Timler Extracts and Essential Oils. *Molecules, 22*(1), 174.
